# Transgenic rhesus monkeys carrying the human *MCPH1* gene copies show human-like neoteny of brain development

**DOI:** 10.1093/nsr/nwz043

**Published:** 2019-03-27

**Authors:** Lei Shi, Xin Luo, Jin Jiang, Yongchang Chen, Cirong Liu, Ting Hu, Min Li, Qiang Lin, Yanjiao Li, Jun Huang, Hong Wang, Yuyu Niu, Yundi Shi, Martin Styner, Jianhong Wang, Yi Lu, Xuejin Sun, Hualin Yu, Weizhi Ji, Bing Su

**Affiliations:** 1 State Key Laboratory of Genetic Resources and Evolution, Kunming Institute of Zoology, Chinese Academy of Sciences, Kunming 650223, China; 2 Primate Research Center, Kunming Institute of Zoology, Chinese Academy of Sciences, Kunming 650223, China; 3 Center for Excellence in Animal Evolution and Genetics, Chinese Academy of Sciences, Kunming 650223, China; 4 Kunming College of Life Science, University of Chinese Academy of Sciences, Beijing 100101, China; 5 Yunnan Key Laboratory of Primate Biomedical Research, Institute of Primate Translation Medicine, Kunming University of Science and Technology, Kunming 650500, China; 6 Department of Psychiatry, University of North Carolina, Chapel Hill, NC 27599-7160, USA; 7 Department of Computer Science, University of North Carolina, Chapel Hill, NC 27599-7160, USA; 8 Key Laboratory of Animal Models and Human Disease Mechanisms, Kunming Institute of Zoology, Chinese Academy of Sciences, Kunming 650223, China; 9 Department of Medical Imaging, the First Affiliated Hospital of Kunming Medical University, Kunming 650032, China; 10 Department of Minimally Invasive Neurosurgery, the First Affiliated Hospital of Kunming Medical University, Kunming 650032, China

**Keywords:** human evolution, brain development, *MCPH1*, transgenic monkey, neoteny, cognition

## Abstract

Brain size and cognitive skills are the most dramatically changed traits in humans during evolution and yet the genetic mechanisms underlying these human-specific changes remain elusive. Here, we successfully generated 11 transgenic rhesus monkeys (8 first-generation and 3 second-generation) carrying human copies of *MCPH1*, an important gene for brain development and brain evolution. Brain-image and tissue-section analyses indicated an altered pattern of neural-cell differentiation, resulting in a delayed neuronal maturation and neural-fiber myelination of the transgenic monkeys, similar to the known evolutionary change of developmental delay (neoteny) in humans. Further brain-transcriptome and tissue-section analyses of major developmental stages showed a marked human-like expression delay of neuron differentiation and synaptic-signaling genes, providing a molecular explanation for the observed brain-developmental delay of the transgenic monkeys. More importantly, the transgenic monkeys exhibited better short-term memory and shorter reaction time compared with the wild-type controls in the delayed-matching-to-sample task. The presented data represent the first attempt to experimentally interrogate the genetic basis of human brain origin using a transgenic monkey model and it values the use of non-human primates in understanding unique human traits.

## INTRODUCTION

Expansion in brain size and improvement in cognitive skills are among the most fundamental evolutionary changes that set humans apart from other primates. Comparative genomic analyses between humans and non-human primates suggest that these dramatic phenotypic divergences may be due to several underlying genetic changes: rapid evolution of protein-coding genes [1,2] and non-coding RNA genes [3–5], emergence of human-specific segmental duplications [6–8], as well as alterations in gene expression [9–12] and epigenetic regulation [13–15]. Despite a great deal of effort in previous studies, we are still on the way in searching for the responsible genes and dissecting the genetic mechanisms that shape the human brain.

Among the reported genes that play important roles in human brain development, *MCPH1* (also known as *BRIT1*) is one of the strong candidates that may contribute to human brain evolution [16]. It is one of the fast-evolving genes in primates [17]. In particular, *MCPH1* has accumulated seven human-specific amino acid changes that are fixed in modern humans [17]. Our previous *in vitro* experiments showed that these human-specific protein-sequence changes could alter the regulation of *MCPH1* on its downstream genes [18]. Importantly, at the transcriptional level, *MCPH1* also has shown human-specific changes. During postnatal brain development, *MCPH1* is abundantly expressed in humans, but less so in non-human primates (macaque and chimpanzee, Supplementary Fig. 1A) [11]. In addition, we have shown that the *MCPH1* transcriptional activity was significantly higher in human than in rhesus monkey [18]. Collectively, current evidence suggests that not only the human-specific protein-sequence changes, but also gene-expression alteration of *MCPH1* may contribute to human brain development and function.

*MCPH1* encodes a pleiotrophic protein. It functions as a transcription factor by interacting with E2F1 (E2F transcription factor 1) to regulate cell cycle and cell apoptosis [19]. It also works as a DNA-damage response protein and is involved in chromatic remodeling to control DNA repair [16,20,21]. In the central nervous system, as a centrosome protein, MCPH1 plays a conserved role in neurogenesis by regulating the neuronal progenitor divison mode via the Chk1–Cdc25B pathway [22]. In humans, truncated mutations of *MCPH1* cause primary microcephaly (MCPH, OMIM251200)—a rare human brain-developmental disorder, characterized by significantly reduced brain volume and mental retardation [23–25]. Consistently, the *MCPH1* knockout animal models (mouse and monkey) reproduced the phenotypes of human microcephaly, notably the reduced brain size [22,26]. During human brain development, *MCPH1* has the highest expression at the prenatal stage and the expression reduces after birth and remains at a constant level through adulthood (Supplementary Fig. 1B). At the prenatal stage, *MCPH1* is highly expressed in all cell types in the cortex, including neural progenitor cells, inter-neurons, astrocytes and microglia cells [27] (Supplementary Fig. 1B). In a mouse study, it was demonstrated that *MCPH1* controls precise mitotic spindle orientation and regulates the progenitor division mode to maintain brain size [22]. However, although *MCPH1* loss of function causes abnormal brain development, resulting in a reduced brain size in human and animals, the functional consequence of the human-specific seqence and expression changes remains to be understood.

To interrogate the genetic basis of human brain evolution, the traditional mouse or rat models are less ideal due to the vast dissimilarities in brain size and structure between humans and rodents. Instead, a non-human primate transgenic model would be far more effective. The rhesus monkey (*Macaca mulatta*), an Old World Monkey species widely used for biomedical research, is an ideal choice, due to its high sequence similarity with humans (>93% for protein-coding genes) [28] and yet relatively large phylogenetic distance (about 25 million years of divergence from humans), which alleviates ethical concerns [29].

For *MCPH1*, the coding sequence similarity is 94.9% between human and rhesus monkey, while it is only 67.5% between human and mouse. Similarly, the 5’ non-coding sequence (∼5 kb) of *MCPH1* likely contains regulatory elements for gene-expression regulation and it has 88.7% similarity between human and rhesus monkey, while it is only 40.4% between human and mouse. Additionally, we have shown that, during primate evolution, the *MCPH1* promoter region has acquired a primate-specific E2F1-binding motif that is absent in rodents and other mammalian species [30]. Taken together, a rhesus monkey model is promising to study the functional impact of the human-specific changes (protein sequence and gene expression) on human brain evolution.

In this study, to mimic the human-specific genetic changes, using lentivirus transfection, we introduced the human *MCPH1* copies (huMCPH1) into the rhesus monkey genome so that the transgenic (TG) monkeys have an overexpression of human *MCPH1*. We successfully generated eight first-generation (F0) and three second-generation (F1) TG monkeys carrying human *MCPH1* copies. Brain-development tracking via magnetic resonance imaging (MRI) of a tissue section with cellular markers showed that the TG monkeys experienced delayed neuronal maturation and neural-fiber myelination, both of which are human-like features of brain-developmental neoteny. Accordingly, transcriptome analysis of prenatal and postnatal brain development revealed an altered gene-expression profile in neuro-progenitors and neurons with shifted expression time of synapse-related genes in the TG monkeys. Remarkably, our preliminary cognitive test detected an improved short-term memory in the TG monkeys.

## RESULTS

### Generation of transgenic monkeys carrying human *MCPH1* copies

All animal procedures were conducted following the international standards, and were approved in advance by the Institutional Animal Care and Use Committee of Kunming Institute of Zoology, Chinese Academy of Sciences and Yunnan Key Laboratory of Primate Biomedical Research (Approval No: SYDW-2010002 and KBI_K001115033-01,01). Lentivirus delivery was used to introduce the human *MCPH1* copy (huMCPH1) into the rhesus monkey genome. A high titer (>1 × 10^10^ infection particles per ml) simian immunodeficiency virus (SIV) vector containing lentivirus was produced for gene transfer (Supplementary Fig. 1C; see the ‘Methods’ section for more details). The human *MCPH1* gene was cloned into the SIV vector containing an eGFP (enhanced green fluorescent protein) gene copy and a universal promoter (the CMV-enhanced chicken beta actin (CAG) promoter) (Supplementary Fig. 1D). The monkey oocytes were obtained by super-ovulation and fertilized *in vitro* (IVF). The early-cleavage-stage embryos were injected with 50–100 pl lentivirus.

**Figure 1.**
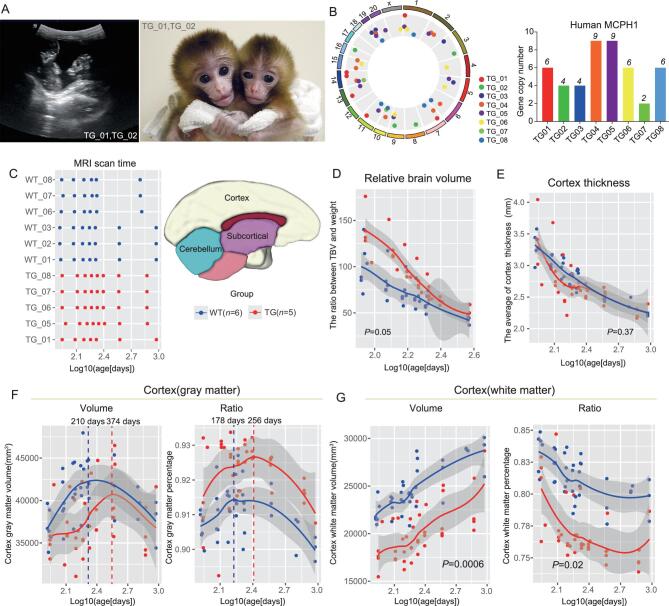
Brain-developmental tracking of the TG monkeys via structural MRI. (A) Left panel: the ultrasound image showing the twin monkeys (TG_01 and TG_02) at 58 days of gestation. Right panel: the newborn twin TG monkeys: TG_01 (male, left) and TG_02 (female, right). (B) Left panel: genomic distribution of the huMCPH1 transgene copies in the TG monkeys. The transgene insertion sites (dots) are randomly distributed on the chromosomes (outermost circle). Right panel: bar plot of the huMCPH1 copy numbers in the TG monkeys. (C) Left panel: the time points (log10(age-days)) of MRI scans of the five TG and six WT monkeys, with the first scan at about 2 months after birth and the last scan at 2–3 years old. Right panel: the schematic map of brain regions. (D) The change in relative brain volume (measured by the total brain volume divided by the body weight) during development. (E) The change in cortex thickness during development. (F) The change in cortex gray-matter volume and ratio during brain development. (G) The change in cortex white-matter volume and ratio during brain development. Group effect *P*-value was calculated based on LMM (linear mixed model) and *P* < 0.05 was taken as statistically significant. The dashed vertical lines indicate the peaks of cortex gray-matter volumes or ratios.

In total, five pregnant surrogates produced eight F0 monkeys (T_01–T_08), among which six are twins (T_01/T_02, T_03/T_04 and T_06/T_07) (Fig. 1 and Table 1). Caesarean section was used to deliver baby monkeys at around 155 days’ gestation except for the twins (T_03 and T_04) with premature abortion at embryonic 136 days. Multiple tissues (blood, placenta, umbilical cord endothelial cells and skin) were sampled to test the transgenic status and all monkeys turned out to be positive (Supplementary Fig. 1E–P). We detected strong GFP signals in the nucleus of the TG monkeys (Supplementary Fig. 1P). Since *MCPH1* is known to work in the nucleus [31], this result suggests that the huMCPH1 transgenes were correctly positioned in the cell.

**Table 1.** Information of the generated transgenic monkeys in this study.

Monkey ID	Generation	Sex	Date of birth	Method of delivery	Status	huMCPH1 copy number
**TG_01**	F0	Male	2011/6/15	C-section	Live	6
**TG_02**	F0	Female	2011/6/15	C-section	Live, deceased at 76 days after birth	4
**TG_03**	F0	Male	–	C-section	Abortion at embryonic 136 days	4
**TG_04**	F0	Male	–	C-section	Abortion at embryonic 136 days	9
**TG_05**	F0	Male	2015/6/18	C-section	Live	9
**TG_06**	F0	Male	2015/6/26	C-section	Live	6
**TG_07**	F0	Female	2015/6/26	C-section	Live	2
**TG_08**	F0	Female	2015/6/26	C-section	Live	6
**TG_09**	F1	Male	–	C-section	Euthanized at embryonic E76 days	5
**TG_10**	F1	Male	–	C-section	Euthanized at embryonic E92 days	6
**TG_11**	F1	Male	–	C-section	Euthanized at embryonic E92 days	5

To determine the integrated genomic locations and copy numbers, we conducted captured next-generation sequencing according to the reported method [32]. As expected for lentivirus, the huMCPH1 copies were randomly integrated into the monkey genomes. The eight TG monkeys have two to nine huMCPH1 copies (Table 1 and Fig. 1B). Importantly, all integration sites are located in either inter-genic or non-coding regions and presumably will not interfere with the function of the monkey endogenous genes (Supplementary Table 1).

For comparison, we recruited six wild-type (WT) monkeys (Fig. 1C and Supplementary Table 2) and initially they were divided into two groups. The first contained three age-matched monkeys (WT_01, WT_02 and WT_03) raised by their biological mothers. The second WT group contained three age-matched monkeys (WT_06, WT_07 and WT_08) who were separated from their biological mothers 6–25 days after birth and raised by humans under the same conditions as the TG monkeys (Supplementary Table 2). Unfortunately, TG_02 died of unknown cause 76 days after birth. A biopsy did not reveal any organ damage. As mentioned above, TG_03 and TG_04 were abortions at embryonic 136 days. For comparison of brain-tissue section and transcriptome analysis, six additional WT monkeys were sacrificed at the corresponding developmental stages (76 days after birth for WT_04 and WT_05, and embryonic 130–145 days for WT_09, WT_10, WT_11 and WT_12) (Supplementary Table 2). In total, we collected data from 8 TG monkeys and 12 WT monkeys.

### Brain-development tracking using structural MRI suggests a delayed neural maturation

To test whether the integrated huMCPH1 copies influenced the brain development of the TG monkeys, we first performed a non-invasive analysis, namely structural MRI (Philips Achieva 3.0T TX). The tested monkeys included five TG monkeys (TG_01, TG_05, TG_06, TG_07 and TG_08) and six WT monkeys (WT_01, WT_02, WT_03, WT_06, WT_07 and WT_08). The MRI scans were conducted at scheduled intervals (once every month while 2–12 months old and then once every 6 months until 2–3 years old) (Fig. 1C). Both T1-weighted image and diffusion tensor imaging (DTI) data were collected. Using the T1 image data, we calculated the volumes of total brain (TB), cerebellum, lobes and subcortical regions based on the published method and monkey atlas [33] (Supplementary Fig. 2). To rule out the potential influence of feeding types, we first compared the two WT monkey groups (monkey feeding vs human feeding) and we did not detect any difference in volumes (Supplementary Table 3). Therefore, all WT monkeys were grouped together in the following analysis.

In general, the TB volume and body weight of the TG monkeys were smaller than the WT monkeys during early development, likely due to the C-section delivery of the TG monkeys at 155 days of pregnancy—about 1 week earlier than the natural delivery of the WT controls. Also, three of the five TG monkeys were twins, which usually weigh less than single-birth monkeys (all WT monkeys were single-birth). However, this difference became smaller when the monkeys grew older and the TG monkeys eventually caught up with the WT monkeys at about 3 years old (Supplementary Fig. 3A and B). Of note, the relative brain volume (TB volume adjusted by body weight) of the TG monkeys was larger than the WT monkeys during early postnatal development and this difference became invisible when the monkeys grew older (Fig. 1D, linear mixed model (LMM) model, group effect *P* = 0.05), while the cortex thickness was similar between them throughout development (Fig. 1E).

As the brain is mainly composed of cortex gray matter (GM, mostly neurons) and subcortical white matter (WM, mostly glial cells) [34], we next conducted segmentation analyses. Notably, during brain development, the cortex GM volume of the TG monkeys increased more slowly than that of the WT monkeys, and there was on average 164 days’ delay of peak time for the TG monkeys (Fig. 1F). When the cortex was divided into four lobes (frontal, parietal, occipital and temporal lobes), we saw the same pattern in all lobes (Supplementary Fig. 3C–F). Interestingly, the cortex GM ratios (the proportion of the cortex GM volume vs the TB volume) of the TG monkeys were larger than those of the WT monkeys with a similar ratio peak time delay (Fig. 1F, LMM model, group effect *P* = 0.06). By contrast, we did not detect such a delay in the cerebellum or subcortical region (Supplementary Fig. 3G and H).

The developmental pattern of WM was different from that of GM. There were no volume/ratio peaks for the cortex WM and the TG monkeys kept a significantly lower volume and cortex WM ratio than the WT monkeys during development (Fig. 1G, LMM model, group effect *P* = 0.0006) (Fig. 1G, LMM model, group effect *P* = 0.01). By contrast, the WM volume of the subcortical region did not show such a difference and an opposite pattern was seen for the cerebellum, although statistically not significant (Supplementary Fig. 3G and H, group effect *P* = 0.59). Given the observed patterns, we reasoned that the observed brain-developmental changes of the TG monkeys might reflect a delay in cortex development rather than a developmental impairment. Consistently with this view, the cortex GM volume curves of the TG and WT monkeys started to converge 1 year after birth (Fig. 1F) and the same pattern was observed when looking at the curves of the four lobes (Supplementary Fig. 3C–F). In particular, TG_01 and three WT monkeys (WT_01, WT_02 and WT_03) had MRI data at later stages (∼3 years old) and the cortex GM and WM volumes of TG_01 had already caught up with the WT monkeys (Supplementary Fig. 4), supporting the proposed brain-developmental delay.

To further explore the brain-developmental changes of the TG monkeys, we analysed the MRI-DTI data to evaluate the WM properties, growth of brain structures and fiber tracts that connect them [35]. The fractional anisotropy (FA) index was used in characterizing the degree of diffusion directionality and is sensitive to the axon size, density as well as the degree of myelination [36]. Consistently with the observation of brain-volume change, in three types of WM tracts (projection fibers, association fiber and commissural fibers), the TG monkeys exhibited relatively lower FA values compared with the WT monkeys, although statistically not significant (Supplementary Fig. 5A–C and Supplementary Fig. 6). The lower FA values suggest lower levels of myelination. A similar pattern was also observed when looking at the MD (mean diffusivity) values [37] (Supplementary Fig. 7). Hence, the MRI-DTI data indicated a lower myelination level in the TG monkeys, implying a delayed neural-fiber myelination and neural-network maturation, which seem to mimic the known brain-developmental delay (neoteny) of humans [38].

We also checked whether there was a correlation between the number of carried huMCPH1 copies and the brain structural measurements, and we did not find a significant correlation with any measurements including TB volume, cortex volume and cortex thickness, etc. (Supplementary Fig. 8), suggesting that a gene-dosage effect is not obvious.

### Brain-tissue-section analysis indicates delayed neuronal differentiation

**Figure 2.**
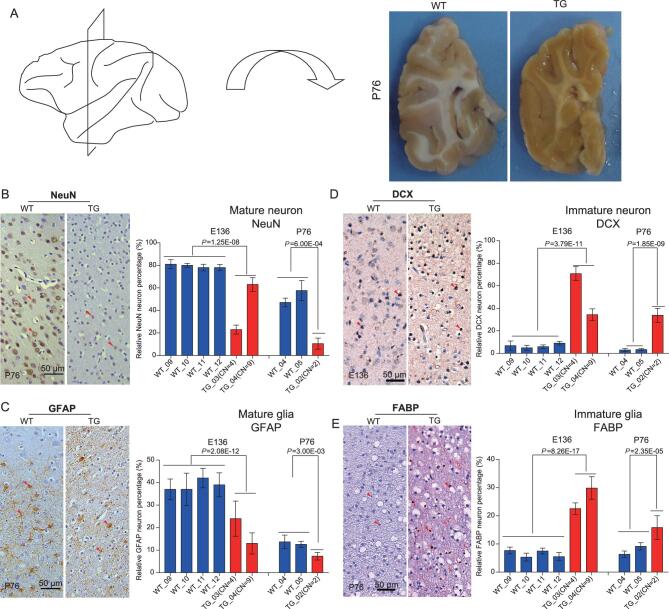
Brain immunohistochemistry analysis with gene markers. (A) The schematic indication of the sampled frontal-lobe region of the P76 monkey brain. (B) Immunohistochemical staining of NeuN, the marker gene for matured neurons. Quantification of the NeuN-positive neurons indicates fewer matured neurons in the TG monkeys compared with the WT monkeys. (C) Immunohistochemical staining of *GFAP*, the marker gene for matured astrocytes. Quantification of the *GFAP*-positive astrocytes indicates decreased mature astrocytes in the TG monkeys. (D) Immunohistochemical staining of *DCX*, the marker gene for immature neurons. Quantification of the *DCX*-positive neurons indicates more immature neurons in the TG monkeys. (E) Immunohistochemical staining of *FABP*, the marker gene for immature astrocytes. Quantification of the *FABP*-positive astrocytes indicates more immature astrocytes in the TG monkeys. All histograms represent the mean ± SD of at least two sections and each section includes counts of four different visual fields. The red arrows indicate positively stained cells. The two-tailed unpaired *t*-test was used for statistical assessment and CN stands for copy number.

To detect brain-developmental changes at the cellular level, we conducted brain-tissue-section analysis of the frontal lobe at both the prenatal (two TG and four WT monkeys at embryonic day 136) and postnatal (one TG and two WT monkeys at 76 days after birth) stages (Fig. 2A). Two marker genes were used to examine the status of neural-cell proliferation and differentiation, including NeuN for matured neurons and glial fibrillary acidic protein (GFAP) for matured astrocytes. At the prenatal stage (E136), there were 80% NeuN-positive cells in the WT monkeys, contrasting with only 20% in TG_03 and 60% in TG_04 (*P* = 1.25E-08, two-tailed *t*-test; Fig. 2B). At the postnatal stage (P76), 40–60% of cells were NeuN-positive in the WT monkeys, but only 10% in TG_01 (*P* = 6.0E-04, two-tailed *t*-test; Fig. 2B). We detected similar ratio differences for astrocytes, with the WT monkeys having twice the ratio of GFAP-positive cells compared with that of the TG monkeys (*P* < 0.01, two-tailed *t*-test; Fig. 2C).

The reduced ratios of matured neurons and astrocytes in the TG monkeys would predict elevated ratios of immature cells. To test this, we used two additional makers, namely DCX (doublecortin) for immature neurons and fatty acid binding protein (FABP) for immature astrocytes. As expected, the TG monkeys possessed much higher ratios of immature neurons and glia cells compared with the WT monkeys (*P* < 0.001, two-tailed *t*-test; Fig. 2D and 2E). Of note, the total numbers of cells in the brain were similar between the TG monkeys and the WT monkeys (Supplementary Fig. 9). Collectively, this cell-level difference is consistent with the observed myelination delay of the MRI data, as the fiber tracts are mostly composed of glial cells and myelinated nerve cells (axons).

### Brain-transcriptome analysis using bulk tissue

To gain insight into the molecular mechanism underling the speculated cortex-developmental delay, we conducted RNAseq of the prefrontal cortex of the prenatal (two TG vs four WT at E136) and postnatal monkeys (one TG vs two WT at P76), with liver and muscle as the references. As expected, the overall *MCPH1* expression was much higher in the TG monkeys than in the WT monkeys for all tissue types and the integrated huMCPH1 copies had much higher expression than the endogenous monkey MCPH1 (Supplementary Figs 10A and 11A). In the brain, there was a large number of differentially expressed genes (DEGs) between the TG and the WT monkeys (970 genes at embryonic day 136 and 1933 genes at postnatal day 76) (Supplementary Figs 10B, C, 11B and C and Supplementary Table 4). The numbers of DEGs were comparable in muscles and much lower in livers. Only a small portion of DEGs overlapped among tissues (Supplementary Figs 10D and 11D and Supplementary Table 4), implying that the transgene huMCPH1 affects the gene expression of the TG monkeys in a tissue-type-dependent manner.

To see the functional enrichment of the DEGs in the brain, we performed gene ontology (GO) ontology analysis using the ToppGene Suite [39]. At the prenatal stage (E136), there were 350 significantly enriched functional categories for the TG-down-regulated genes (Supplementary Table 5, FDR B&H < 0.05) and the top four categories were all related to synaptic signaling (Supplementary Fig. 10E, left panel). In contrast, the enriched categories for the TG-up-regulated genes (60 categories, Supplementary Table 6) were mostly basic cellular functions such as translation and protein localization to the endoplasmic reticulum, not closely related to neural function (Supplementary Fig. 10E, right panel). Similarly, at the postnatal stage (P76), the top 10 enriched categories for the TG-down-regulated genes were all related to neuron differentiation and neuron development, and synaptic-signaling genes were also over-represented, while the top categories for the TG-up-regulated genes were mostly related to neuron projection (Supplementary Fig. 11E and Supplementary Table 7). The transcriptomic changes in the developing brains suggest that many neuron-maturation and differentiation-related genes were suppressed in the TG monkeys, consistently with the observed delay of neuron differentiation in the tissue-section analysis (Fig. 2).

**Figure 3.**
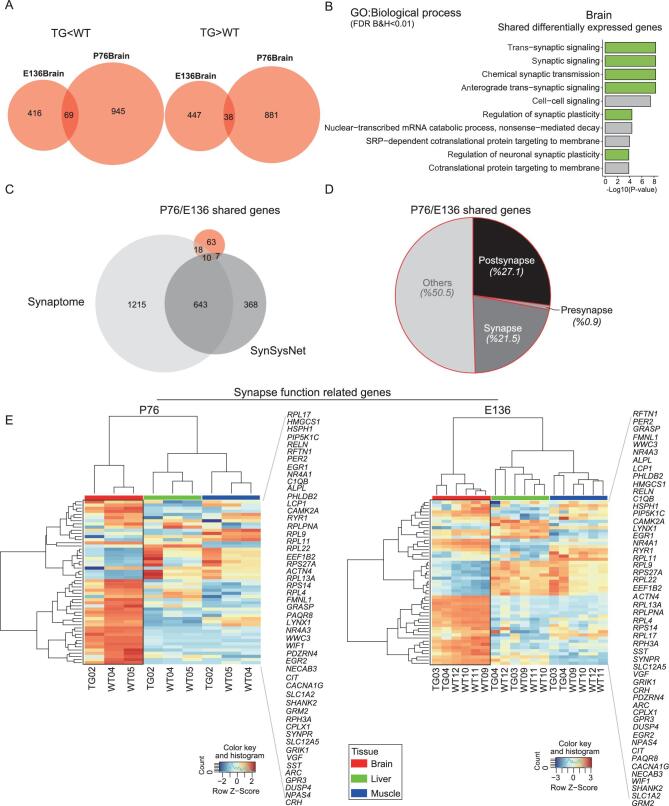
Brain-transcriptome analysis at prenatal E136 and postnatal P76. (A) Venn diagrams showing overlaps among differentially expressed genes (DEGs, TG vs WT) in the brain between E136 and P76. (B) Enriched gene clusters of DEGs in the brain. The green blocks indicate synapse-signaling-associated clusters. (C) Venn diagrams showing overlaps among the E136/P76 shared genes, the synaptome genes and the synsysnet genes. The hypergeometric tests indicate significant overlaps with the synaptome genes (*P* = 4.89E-08) and with the synsysnet genes (*P* = 7.74E-06). (D) Pie chart of the E136/P76 shared genes indicating that about 50% of the genes are synapse- and post-synapse-related genes. (E) Hierarchical clustering using the E136/P76 shared brain DEGs.

Importantly, we found 107 brain DEGs shared between the prenatal E136 and postnatal P76 stages (Fig. 3A). GO ontology analysis with these 107 genes indicated that the highest enrichment category was synapse-related function, confirming the observed pattern when using all brain DEGs (Fig. 3B, 6/10). Remarkably, 35 of the 107 genes (32.7%) overlapped with the known synapse genes in the datasets of *synaptome* [40] and *synsysnet* [41] (Fig. 3C). Further analysis showed that about 50% of the 35 genes are either post-synapse- or synapse-related genes, and only 0.9% are pre-synapse-related genes (Fig. 3D). The hierarchical clustering analysis using the shared brain DEGs clearly distinguished the TG and the WT monkeys with the most prominent distinction between brain and muscle/liver (Fig. 3E). For example, *NR4A1* is a downstream gene of *MEF2A*—a gene playing a critical role in activity-induced synaptic modification [42]. This gene showed 76% (at E136) and 26% (at P76) expression reduction in the TG monkeys. In the mouse model, overexpression of *NR4A1* would eliminate dendritic spines while knock-down of *NR4A1* could cause excessive number of spines and major postsynaptic density [43]. Together, these data suggest that, at the bulk-tissue level, the transgene huMCPH1 mainly suppresses the expression of neural differentiation and synapse-function-related genes.

### Generation of F1 TG monkeys and transcriptome analysis of fetal cortical lamina

**Figure 4.**
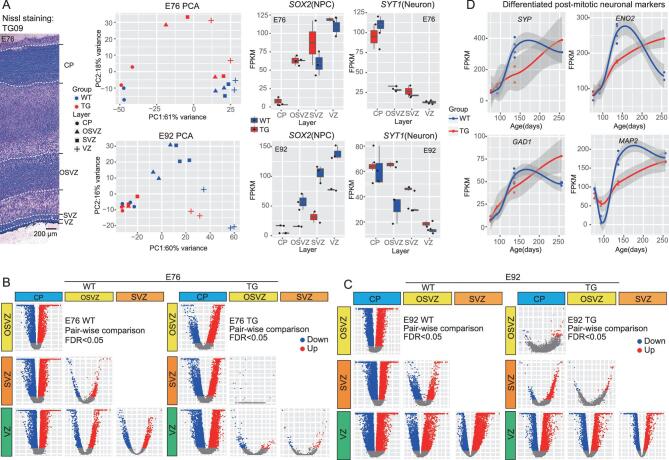
Transcriptome analysis of cortical laminae at E76 and E92. (A) Laser micro-dissection of cortical-laminae and RNAseq analysis. Left panel: Nissl staining of the E76 cortex (TG-09) showing the cortical laminae. Middle panel: the PCA maps of E76 and E92 based on expression levels of all genes. Right panel: cell-marker analysis with SOX2 for neuro-progenitor cell and SYT1 for neuron. (B) Volcano plots showing pairwise comparisons of gene expression between the indicated laminae of E76. (C) Volcano plots showing pairwise comparisons of gene expression between the indicated laminae of E92. (D) Four mature neuron gene makers showing expression delay in the TG monkeys.

To further dissect the impact of the huMCPH1 copies on brain development, we generated three F1 TG monkeys by IVF using the sperms of TG_01 that we proved were carrying the huMCPH1 copies in the germ line (Supplementary Fig. 12). The three F1 TG monkeys were sacrificed at embryonic day 76 (TG_09 at E76) and embryonic day 92 (TG_10 and TG_11 at E92)—the two developmental time points during the neurogenesis peak in rhesus monkeys [44]. Captured sequencing analysis indicated that the three F1 TG monkeys all carried the huMCPH1 copies at the same integrated sites as TG_01 (Supplementary Table 1). With the use of IVF, we also obtained five WT fetal monkeys at the corresponding developmental points (two WT at E76 and three WT at E92, Supplementary Table 2). To conduct more detailed developmental tracking, we sampled the frontal cortex and dissected (using laser micro-dissection) the brain tissue into four cortical laminae, as they reflect different stages of neural proliferation, differentiation and migration, including the cortical plate (CP), outer sub-ventricular zone (OSVZ), sub-ventricular zone (SVZ) and ventricular zone (VZ) (Fig. 4A). RNAseq was performed for each lamina.

First, we checked *MCPH1* expression in WT monkey embryos and we found that, at E76, no difference existed between the CP and the germinal zone (OSVZ, SVZ and VZ), while, at E92, *MCPH1* expression was higher in the germinal zone than in the CP, consistently with the reported pattern in mouse [24] (Supplementary Fig. 13A). As expected,

in the TG monkey embryos, the transgene huMCPH1 had a much higher expression than the endogenous *MCPH1* in all laminae at both E76 and E92 (Supplementary Fig. 13B). The fold changes were different among different laminae. SVZ had the highest fold change at E76, while CP had the highest fold change at E92 (Supplementary Fig. 13B).

The principal component analysis (PCA) showed that the RNA profiles can distinguish different developmental cortical laminae. The VZ and CP were clearly separated, as they represent undifferentiated neuro-progenitors and differentiated neural cells, respectively, while the separation between the SVZ and OSVZ was incomplete because they are the intermediate stages in view of cell proliferation, migration and differentiation (Fig. 4A). Consistently, the marker genes for neuro-progenitors (*SOX2*) and neurons (*SYT1*) showed the expected expression pattern in these laminae (Fig. 4A). We then analysed DEGs of the four laminae between the TG monkeys and the WT monkeys. As expected, the transgene huMCPH1 caused expression changes of many genes and this pattern was more pronounced at E92 compared with E76 (140–350 genes for E76 and 3000–9000 genes for E92, Supplementary Table 8) (Supplementary Fig. 13C and D). The GO ontology analysis showed that, at E76, there were 25 enriched categories for CP, 26 for OSVZ, 41 for SVZ and 10 for VZ (Supplementary Tables 9–12, FDR B&H < 0.05). Among the top 10 categories, the enriched functional terms were cell development (CP, 5/10), synapse signaling (OSVZ, 5/10), cell differentiation and proliferation (SVZ, 5/10) and cilium function (VZ, 3/10) (Supplementary Fig. 13E, left panel, FDR B&H < 0.05). In contrast, there were a lot more enriched categories at E92 (453 for CP, 109 for OSVZ, 102 for SVZ and 786 for VZ) (Supplementary Tables 13–16, FDR B&H < 0.05), among which the top categories were mRNA catabolic process (CP, 3/10), neuron differentiation, neurogenesis and cell migration (OSVZ, 5/10), cell cycle and mRNA processing (SVZ, 10/10) and immune response (VZ, 8/10) (Supplementary Fig. 13E, right panel, FDR B&H < 0.05).

We next conducted lamina-to-lamina pairwise comparisons in the TG and the WT monkeys separately. Markedly, at E76, the TG monkeys exhibited much less between-lamina expression difference than the WT monkeys. In particular, there were no DEGs when comparing SVZ with OSVZ in the TG monkeys, contrasting with 557 DEGs in the WT monkeys (Fig. 4B and Supplementary Table 17). A similar pattern was seen at E92. For example, there were 61 CP vs OSVZ DEGs in the TG monkeys, while there were 9186 DEGs in the WT monkeys (Fig. 4C and Supplementary Table 18). This result suggests that cortical-lamina distinction is much weaker for the TG monkeys compared with the WT monkeys, supporting the proposed delay of neuronal maturation and differentiation. Consistently, we observed delayed expression peaks of four known neuron-differentiation markers, including *SYP* (Synaptic vesicle protein p38), *ENO2* (Cytosolic protein t), *GAD1* (Glutamic acid decarboxylase) and *MAP2* (Cytoskeletal protein) (Fig. 4D).

Furthermore, in order to see the temporal pattern of gene-expression delay in the TG monkeys, we combined the RNAseq data of all four developmental stages, including E76, E92, E136 and P76 (6 TG vs 11 WT). We classified those genes showing expression delay in the TG monkeys into three types according to their expression peak times in the WT monkeys (Supplementary Fig. 14A and B). The Type-1 genes are those with an expression peak shift from E92 in the WT monkeys to E136 in the TG monkeys (e.g. the *SLC44A2* gene). The Type-2 genes had a peak shift from E136 in the WT monkeys to P76 in the TG monkeys (e.g. the *SYP* gene), while the Type-3 genes had a peak shift from E92 in the WT monkeys to P76 in the TG monkeys (e.g. the *CDK5* gene) (Supplementary Fig. 14A). In total, we identified 185, 347 and 50 genes for Type-1, Type-2 and Type-3 delays, respectively (Supplementary Fig. 14B). We then performed GO ontology analysis and only the Type-2 genes showed significant enrichment of functional categories, implying that the developmental stage close to birth was the most affected in the TG monkeys. Consistently with the results of bulk-tissue RNAseq, the Type-2 genes from the cortical laminae containing undifferentiated cells (VZ, SVZ and OSVZ) were mainly enriched for synapse-related functions such as trans-synapse signaling, chemical-synapse transmission and synaptic signaling (Supplementary Fig. 14C, *P* < 0.001) and the involved genes showed delayed expression peaks at P76 or later stages in the TG monkeys.

To test whether the observed gene-expression delay in the TG monkeys show human-like features, we obtained data from a previous study in which genes with human-specific expression delay were identified by comparing postnatal brain development in the prefrontal cortex of humans, chimpanzees and rhesus macaques [11]. We found that only the Type-2 genes were enriched in the reported Module-I gene set with human-specific expression delay (*P* < 0.0001; hypergeometric test; Supplementary Fig. 14D and Supplementary Table 19). For example, *MEF2A* is a Type-2 gene and also a Module-I gene, which not only plays a role in neuron differentiation [45], but also mediates a human-specific time shift of cortex synaptic development [11]. Hence, the patterns of gene-expression delay are consistent between the data from the bulk tissue and the data for the laminae, and many neural-differentiation-related genes were suppressed in the TG monkeys with human-like expression delays during brain development.

### General behavior analysis and test of short-term memory

To test whether the observed brain-developmental delay at the molecular and cellular levels in the TG monkeys can be transformed into cognitive changes, we first performed an analysis of general behaviors (four TG vs four WT age-matched monkeys 24–36 months old; see the ‘Methods’ section for details). A total of nine indexes for general behaviors were measured [46], including self-injury behavior, stereotypical behavior, feeding, self-grooming, locomotion, resting, bouts of wake, wake and sleep. No difference was detected between the TG and the WT monkeys, suggesting that the transgene huMCPH1 did not cause abnormal behaviors in the TG monkeys (Supplementary Fig. 15).

**Figure 5.**
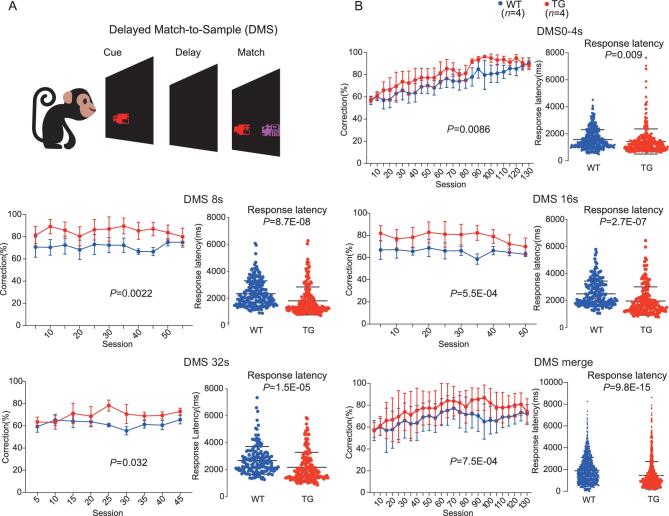
Test of short-term memory using the DMS task. (A) Schematic diagram of the DMS task. (B) The results of the DMS trials indicating different performances between the TG and WT monkeys at different delay times including 0–4, 8, 16 and 32 sec. The bottom-right panel presents the merged DMS data of all delay times. The group effect *P*-values for correction percentage comparison were computed based on the general linear model (GLM) and corrections for multiple tests were conducted using Bonferroni. The *P*-values for reaction time were calculated using the two-tailed Student’s *t*-test.

Next, we performed a test of short-term memory using the delayed-matching-to-sample (DMS) task, which was known to be correlated with prefrontal-cortex function [47–49]. The computerized touch-screen behavioral battery (Cambridge Neuropsychological Test Automated Batteries, CANTAB; Lafayette, USA) was used. The DMS task requires the monkeys to remember the color and the shape of a stimulus on the screen for a specified delayed time. The monkeys were initially habituated in a testing room for 5 days and then subject to touch training. Touch training was divided into two phases. In Phase-1, monkeys were subject to touch training for 15 days continuously. In Phase-2, monkeys were required to have a >85% correction rate for 3 continuous days in the training sessions and then were subject to the DMS task test (Fig. 5A, Supplementary Fig. 16 and Supplementary Video 1; more details about touch training is provided in the ‘Methods’ section). The results showed that, at 0 ∼ 4-sec delayed times, the TG monkeys performed significantly better than the WT monkeys (Fig. 5B, general linear model (GLM) model, group effect *P* = 0.0086) and this difference became more pronounced in the sessions with 8 and 16-sec delayed times (Fig. 5B; GLM model, 8 s, group effect *P* = 0.0022; 16 s, group effect *P* = 5.5E-04). When the delayed time extended to 32 sec, the difference remained (Fig. 5B, GLM model, group effect *P* = 0.032). When all sessions with different delayed times were combined together, the TG monkeys had a significantly better performance than the WT monkeys (Fig. 5B, GLM model, group effect *P* = 7.5E-04). Interestingly, we also observed significantly shorter reaction times (response latency) in the TG monkeys for all categories of delayed times (*P* < 0.001, two-tailed *t*-test, Fig. 5B). Collectively, the TG monkeys exhibited better performance in the DMS task than the WT monkeys, suggesting that the brain-developmental delay caused by the transgene huMCPH1 may have enhanced the short-term memory of the TG monkeys.

## DISCUSSION

*MCPH1* is one of the strong candidates for human brain evolution, since it has accumulated a human-specific protein-sequence and gene-expression changes [17,18]. Ideally, a gene-replacement model would be preferred so that the influence of the endogenous monkey *MCPH1* could be removed. However, due to the long generation time of monkeys (4–5 years), current gene-editing tools are still impractical in generating such a model in monkeys. We argue that a transgenic monkey model is practical and to a large extent can mimic the human-specific status. The transgenic monkeys carry the human *MCPH1* copies so that the effect of the human-specific protein-sequence changes can be tested. At the same time, since the transgenic monkeys over-express the huMCPH1 transgene, this can mimic the human-specific increase in gene expression.

Because *MCPH1* is a key gene for neurogenesis, one of the expected phenotypic outcomes in the transgenic monkeys would be a larger brain, which was not the case in this study. We showed that the TG monkeys carrying the huMCPH1 transgene did not manifest an enlarged brain size, implying that a single gene likely has a limited effect on neural progenitor pool proliferation during brain development. Alternatively, it is equally possible that the human-specific changes in *MCPH1* may not enhance its known function in neuro-progenitor proliferation [22]; rather, they work on the unknown function of *MCPH1* in neuronal maturation, neural plasticity and synapse signaling, which were supported by multiple lines of evidence presented in this study.

Our analyses found a developmental delay of GM in the brain of the TG monkeys, suggesting that the huMCPH1 transgene may delay neuron differentiation and maturation during brain development. Consistently with the cortex-developmental delay, there were much fewer mature neurons and glia cells in the TG monkeys compared with the WT monkeys during the early period of postnatal development. Consistently, the tissue-level transcriptome comparisons indicated a large amount of neuron differentiation and development, and synapse genes were suppressed in the TG monkeys, providing a possible molecular basis for the observed delay in cell maturation and fiber myelination in the brain. In fact, our previous *in vitro* experiments demonstrated that MCPH1 can act as a transcription repressor and repress telomerase activity [50]. Furthermore, the cortical-lamina transcriptome comparisons showed that the huMCPH1 transgene can influence gene expression at all laminae including the CP, OSVZ, SVZ and VZ as early as prenatal E76, indicating that the huMCPH1 transgene may alter neurogenesis by affecting the neuro-progenitor cell division and differentiation. In fact, previous mouse studies have already shown that MCPH1 is required for precise mitotic spindle orientation during neurogenesis [22]. Additionally, as expected, cortical-lamina pairwise comparisons suggested that the between-laminae differences in the TG monkeys were not as obvious as in the WT monkeys, consistently with the proposed delay in neural differentiation. Hence, the huMCPH1 transgene may have contributed to delaying cortical-lamina differentiation in the TG monkeys. Taken together, we propose that overexpression of the huMCPH1 transgene can cause neural-developmental delay due to the down-regulation of many neural-differentiation-related genes. Future experiments are warranted to reveal the detailed molecular pathways.

One hallmark difference between humans and non-human primates is that humans require a much longer time to shape their neuro-networks during development, greatly elongating childhood, namely the so-called ‘neoteny’. Myelination is the process of generating myelin sheaths around nerve fibers so that neural signals can be propagated more swiftly with less signal loss. This process is considered a key developmental aspect of the human brain and continues for at least 10–12 years after birth, providing an extended window of neural-network plasticity [51]. In fact, human neocortical myelination is developmentally protracted compared with that of chimpanzees [52]. We speculate that the observed neural-maturation delay in the TG monkeys may have extended their time window of neural-network plasticity, similar to the brain-developmental neoteny of humans. In support of our speculation, when we combined the RNAseq data of all developmental stages, we found that many of the delay genes were synapse-related genes, which are required for the experience-dependent process of neural-network plasticity [53]. More interestingly, most of the delay genes showed human-specific changes in the timing of synaptic development in the previous study [11]. For example, *MEF2A* and *SYP* were among the key genes showing human-specific delay of youth-like expression compared with chimpanzee and macaque [11]. Notably, synapse and spine density in the human-projection neurons is much higher than that in rhesus macaque, which is associated with the higher cognitive performance in humans [11,54].

The speculated extension of neural-network plasticity in the TG monkeys gained further support from our preliminary cognitive data. The TG monkeys showed an improved short-term memory, suggesting that the observed brain-developmental delay in the TG monkeys is beneficial, possibly through extending the time window of neural-network plasticity. More interestingly, the TG monkeys displayed a significantly shorter reaction time than the WT monkeys during the DMS task, which is another hint at cognitive improvement. More sophisticated cognitive tests are needed to understand the long-term effect of the huMCPH1 transgene in the TG monkeys.

Our findings demonstrated that transgenic non-human primates (excluding ape species) have the potential to provide important—and potentially unique—insights into the basic questions of what actually makes humans unique, as well as into disorders and clinically relevant phenotypes, such as neurodegenerative and social-behavior disorders that are difficult to study by other means [32,46,55]. But such gains must invariably be weighed against potential ethical concerns [29,56,57]. We noted that the transgenic monkey model also has limitations, including the influence of the endogenous monkey gene copy and the incapability to differentiate the effects of protein-sequence changes from gene-expression changes. Several recent technical improvements (e.g. CRISPR-Cas9) have shown the hope of conducting precision genome editing in monkeys [26,58–61], providing more powerful tools for future studies in understanding the genetic basis of human brain evolution.

## METHODS

The detailed methods and materials are available as Supplementary Data at *NSR* online.

## Supplementary Material

nwz043_Supplemental_Files
