# Supplementary material for: Transgenic rhesus monkeys carrying the human MCPH1 gene copies show human-like neoteny of brain development
Source: Natl Sci Rev. 2019 Mar 27;6(3):480–93. doi: 10.1093/nsr/nwz043 (PMC8291473; doi:10.1093/nsr/nwz043)
Supplement: nwz043_Supplemental_Files [file nwz043_supplemental_files.zip › Supplementary data.docx]

**Supplementary Table S2.** Information of wild type monkeys in this study. * MF, monkey feeding; HF, human feeding; Numbers in the parentheses indicate the days of baby monkeys separated from their biological mothers.

| **Monkey ID** | **Monkey Group** | **Sex** | **Birth Date** | **Method of delivery** | **Status** |
| --- | --- | --- | --- | --- | --- |
| WT_01 | WT_MF | Female | 2011-06-18 | Natural birth | Live |
| WT_02 | WT_MF | Male | 2011-06-20 | Natural birth | Live |
| WT_03 | WT_MF | Female | 2011-06-20 | Natural birth | Live |
| WT_04 | WT_HF (16) | Female | 2012-04-07 | Natural birth | Euthanized at 76 days after birth |
| WT_05 | WT_HF (16) | Female | 2012-04-08 | Natural birth | Euthanized at 75 days after birth |
| WT_06 | WT_HF (7) | Male | 2012-03-23 | Natural birth | Live |
| WT_07 | WT_HF (9) | Male | 2012-04-15 | Natural birth | Live |
| WT_08 | WT_HF (25) | Male | 2012-04-18 | Natural birth | Live |
| WT_09 | WT | Female | / | C-section | Euthanized at embryonic 140 days |
| WT_10 | WT | Female | / | C-section | Euthanized at embryonic 130 days |
| WT_11 | WT | Female | / | C-section | Euthanized at embryonic 145 days |
| WT_12 | WT | Male | / | C-section | Euthanized at embryonic 135 days |
| WT_13 | WT | / | / | C-section | Euthanized at embryonic 76 days |
| WT_14 | WT | / | / | C-section | Euthanized at embryonic 76 days |
| WT_15 | WT | Female | / | C-section | Euthanized at embryonic 92 days |
| WT_16 | WT | Male | / | C-section | Euthanized at embryonic 92 days |
| WT_17 | WT | Female | / | C-section | Euthanized at embryonic 92 days |
| WT_18 | WT | Male | 2015-03-08 | Natural birth | Live |
| WT_19 | WT | Female | 2015-06-24 | Natural birth | Live |
| WT_20 | WT | Female | 2015-09-04 | Natural birth | Live |
| WT_21 | WT | Male | 2015-05-25 | Natural birth | Live |

**Supplementary Figure S1**


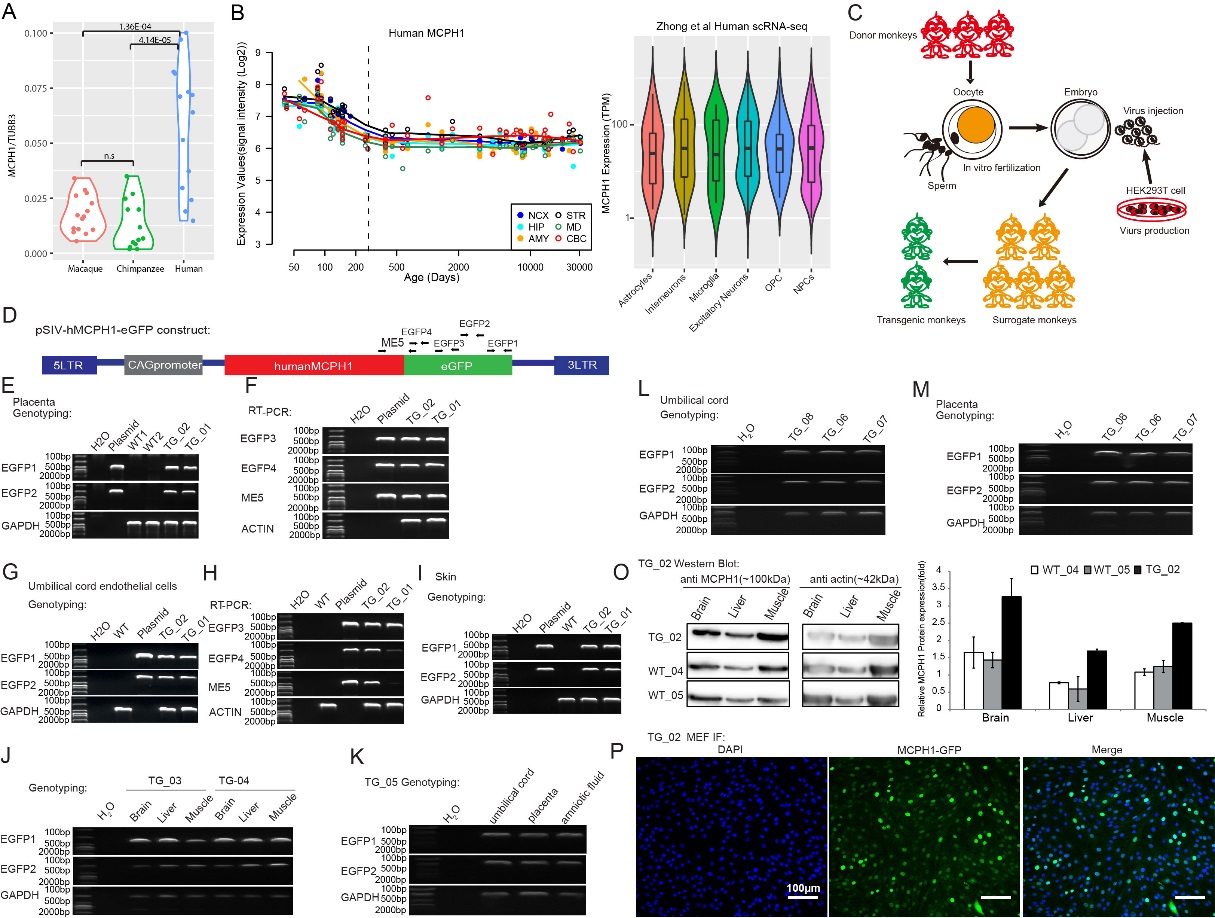


**Figure S1.** **Generation of huMCPH1 transgenic rhesus monkeys.** **(A)**Comparison of MCPH1expression levels (indicated by RPKM values) among human (n=14), chimpanzee (n=13) and macaque (n=15). The MCPH1 expression levels were normalized with the housekeeping gene (TUBB3). Two tailed unpaired *t* test was used. **(B)** Left panel: The curve of MCPH1 expression changes in brain regions including NCX (neocortex), Hip (hippocampus), AMY (amygdala), STR (striatum), MD (mediodorsal nucleus of the thalamus), CBC (cerebellar cortex) during human brain development. The data was downloaded from human brain transcriptome database (<http://hbatlas.org/pages/hbtd> ). Right panel: MCPH1 gene expression pattern in different cell types including astrocytes, interneurons, microglia, excitatory neurons, OPC (oligodendrocyte progenitor cells) and NPC (neuron progenitor cells). **(C)** A schematic map showing the major procedure of generating huMCPH1 transgenic monkeys. **(D)** Structure of the lentiviral vector with an inserted human MCPH1 gene copy next to the eGFP gene copy, which should result in the expression of a fusion protein (huMCPH1-eGFP). Arrows indicate the positions of the primers used for PCR and RT-PCT analyses. eGFP, enhanced green fluorescence protein; LTR, long terminal repeat. **(E-F)** Presence of the transgene huMCPH1 in placenta of the two transgenic monkeys (TG_01 and TG_02) confirmed by PCR (left panel), RT-PCR (right panel). **(G-H)** Results of PCR (left panel) and RT-PCR (right panel) confirmed the presence of huMCPH1 in umbilical cord endothelial cells. **(I)** Presence of huMCPH1 in skin confirmed by PCR. **(J)** PCR results indicating the presence of huMCPH1 in brain, liver and muscle of TG_03 and TG_04. **(K)** PCR results indicating the presence of huMCPH1 in umbilical cord, placenta and amniotic fluid of TG_05. **(L-M)** PCR results indicating the presence of huMCPH1 in umbilical cord and placenta of TG_06, TG_07 and TG_08. **(O)** Western blot analysis confirmed the expression of MCPH1 proteins in brain, liver and muscle tissues (left panel). Quantitative analysis of MCPH1 proteins indicates a higher expression in TG_02 as compared with the two WT monkeys (WT_04, WT_05) (right panel). All data are presented as mean ± sd. The two-tailed unpaired *t*-test was used. **(P)** The nucleus localization of the transgene huMCPH1 in cultured skin cells from TG_02, as indicated by the green fluorescent signals of eGFP.

**Supplementary Figure S2.**

**
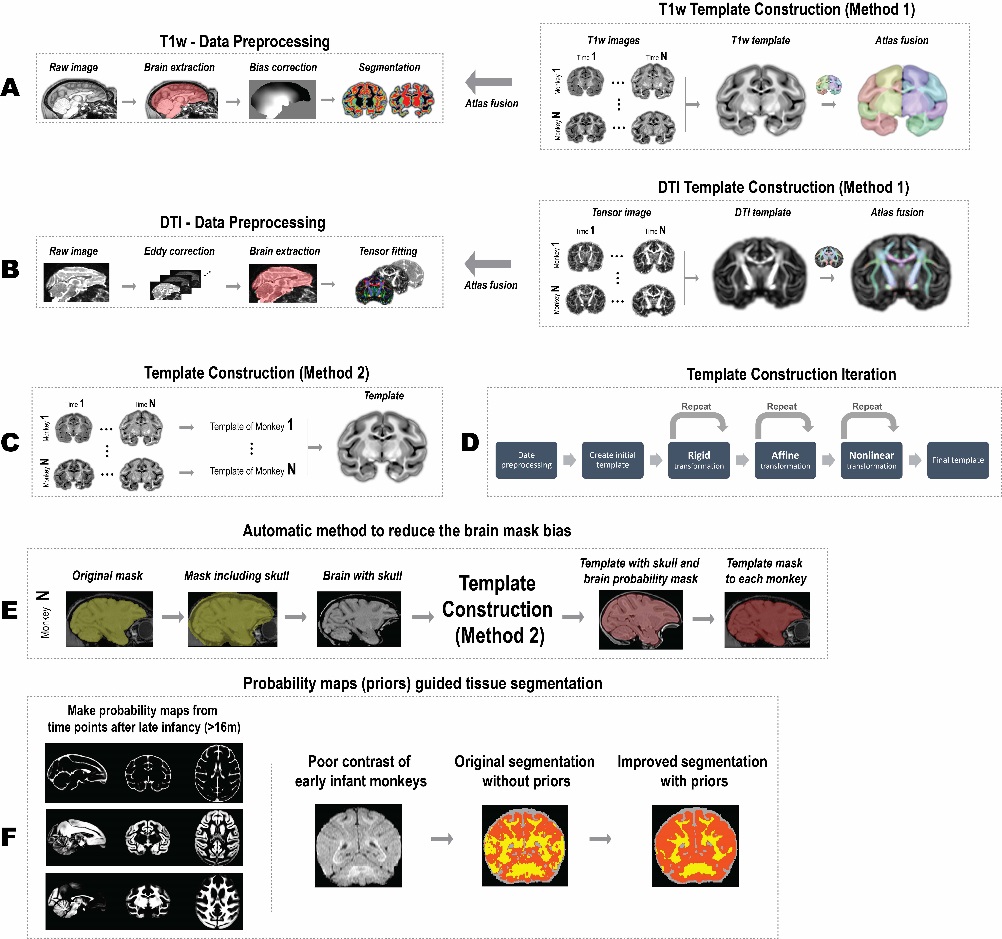
**

**Figure S2. MRI data analysis pipeline. (A)** Data preprocessing of T1w images. Brains were first extracted using a semi-automatic method, after which brains were bias-corrected and segmented into partial volume images of gray matter, white matter and cerebrospinal fluid. All preprocessed T1w images were used to construct a study-specific template using ANTs. The brain parcellation of the infant macaque atlas (Liu et al., 2015) was transformed to a study-specific template, fused into the native space of each scan and used for the analyses of tissue volumes. **(B)** Data preprocessing of DTI. DWI images were eddy-corrected and a brain mask was created for each scan. Diffusion tensor-based measurements were then calculated, including fractional anisotropy (FA), mean diffusivity (MD), axial diffusivity (AD) and radial diffusivity (RD). All tensor images were registered to a study-specific DTI template using the DTI-TK. The white matter ROIs of the UW-DTIRMAC271 atlas (Zakszewski et al., 2014) was transformed to the DTI template, fused into each scan, and used for the analyses of diffusion measurements. **(C)** An alternative method of template construction. Instead of creating a study-specific template using all images, animal-specific templates were first created using all scans from each monkey and then these animal-specific templates were used to generate the final templates. **(D)** All templates were created via an iterative procedure, in which rigid alignments, affine transformation, and nonlinear transformation were repeated until the average image converged for each transformation. Automatic method to reduce human bias of the original brain mask. **(E)** Original masks were dilated to extract brains with parts of skulls. A new template and a brain probability mask were created. The brain probability mask was binarized, manual corrected, and nonlinearly transformed back to the native space of each scan for the final brain extraction. **(f)** Tissue probability maps from later time-points (>16-month) were created and used to guide the segmentation as priors, which effectively improve the segmentation of infant monkey brain.

**Supplementary Figure S3.**

**
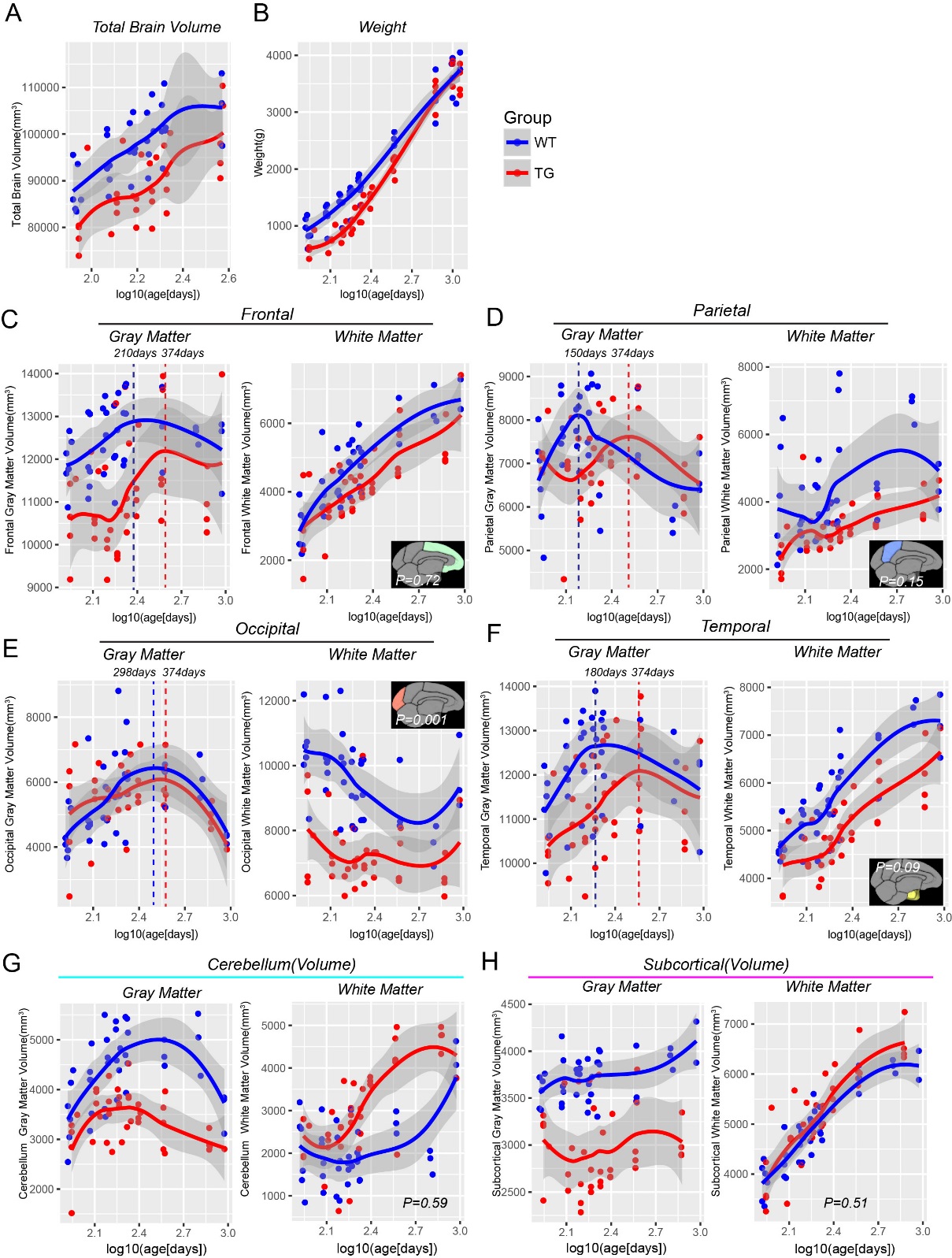
**

**Figure S3. The developmental change patterns of brain volume and body weight. (A)** the change of total brain volume during development; **(B)** the change of body weight during development; **(C)** Frontal lobe; **(D)** Parietal lobe; **(E)** Occipital lobe; **(F)** Temporal lobe. **(G)** The change of cerebellum gray matter and white matter volume during brain development. **(H)** The change of subcortical gray matter and white matter volume during brain development.*P* value was calculated using the linear mixed model, and *p*<0.05 was taken as significant. The dashed vertical lines indicate the peak times of gray matter volume. Blue color stands for the WT and red color stands for the TG monkeys.

**Supplementary Figure S4.**

**
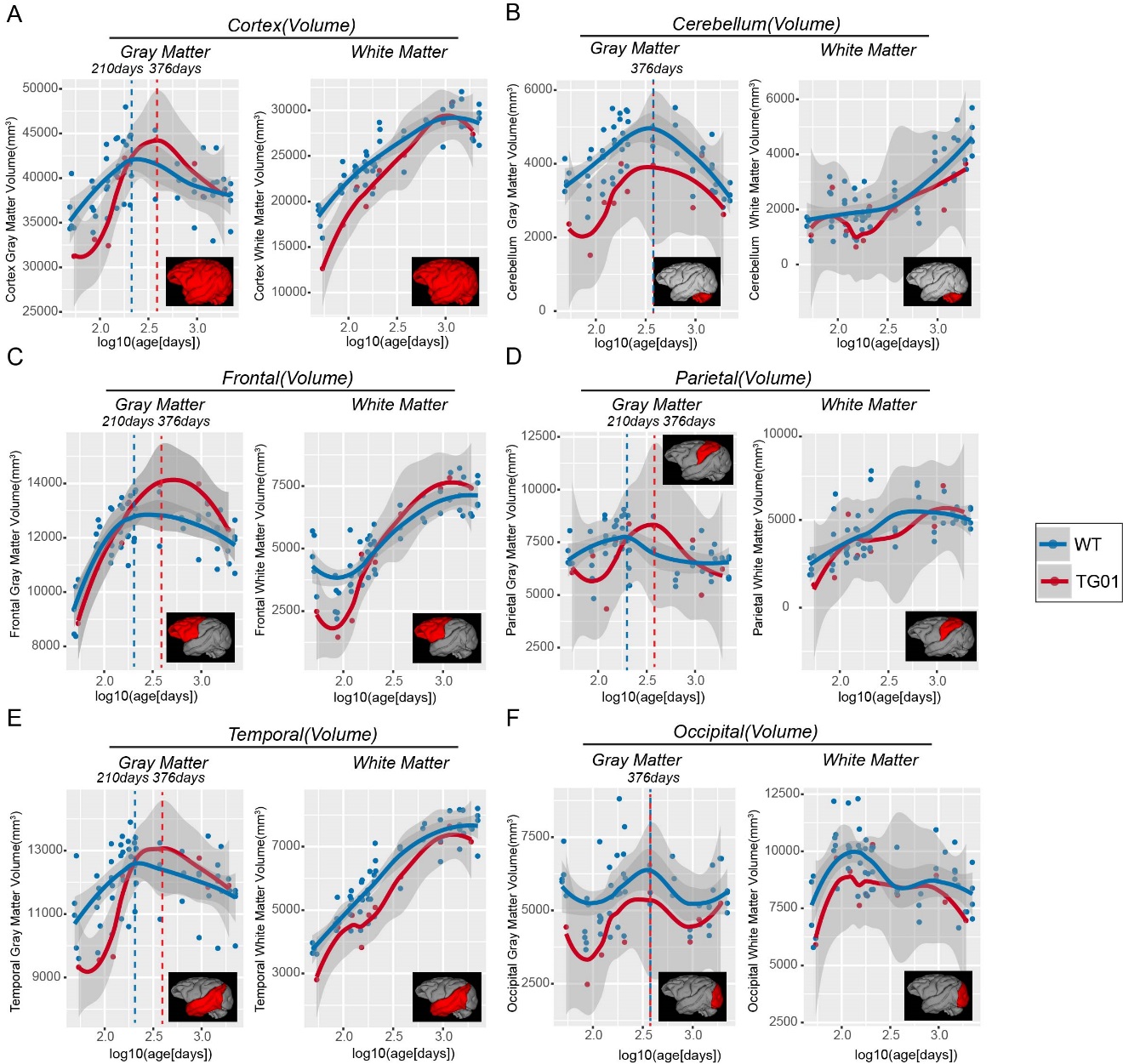
**

**Figure S4. Brain developmental tracking of TG_01 *via* structural MRI.** (**A-B**) Cortex and cerebellum gray matter and white matter volume changes during brain development. (**C-F**) Gray matter and white matter volume changes of the four lobes during brain development. The dashed vertical lines indicated the peak times of gray matter volume. Blue color stands for the WT and read color stands for the TG monkeys.

**Supplementary Figure S5.**


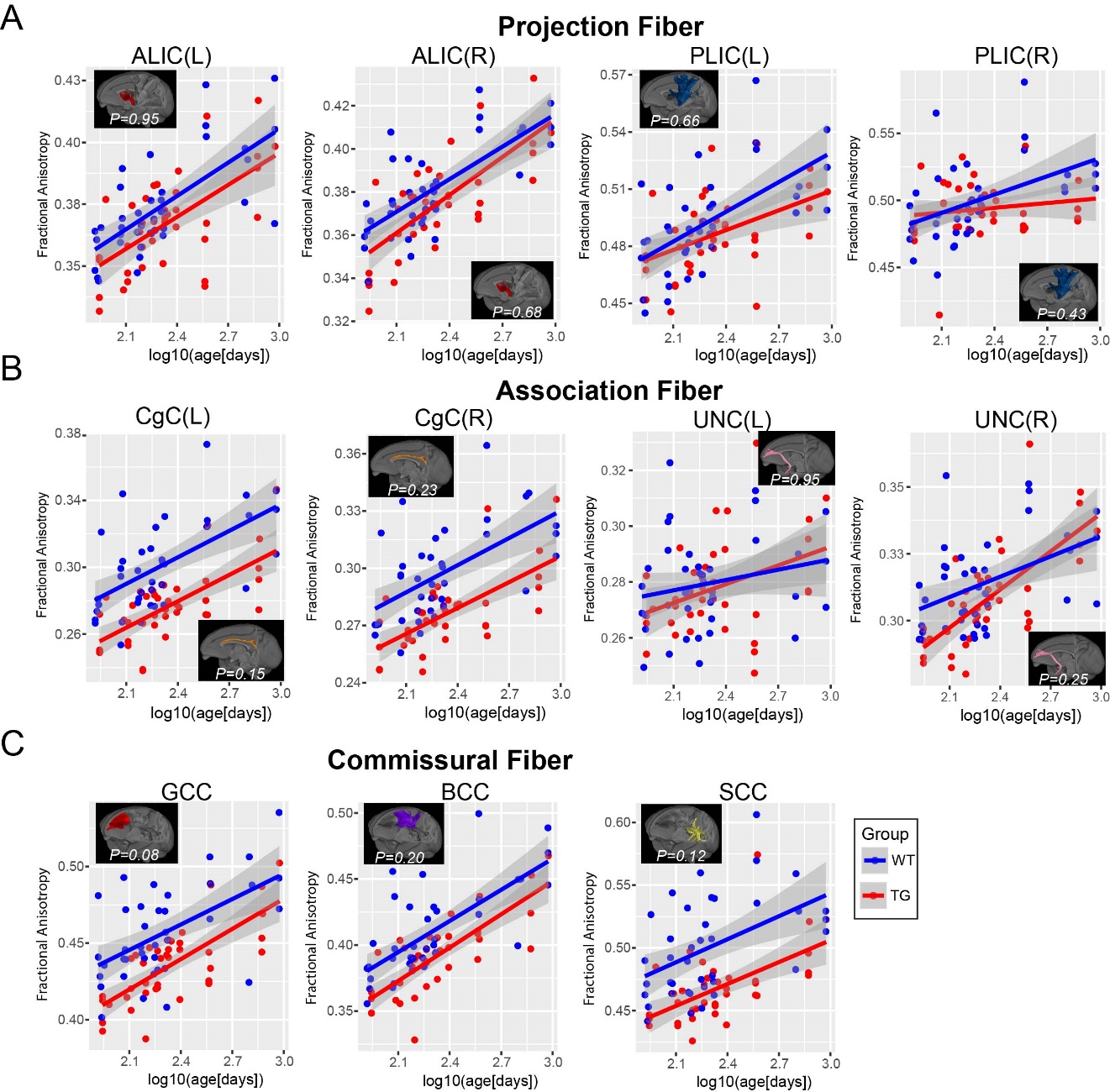


**Figure S5. Brain developmental tracking of neural fiber myelination reflected by the FA (fractional anisotropy) values of WM fibers**. The fibers include: **(A)** The projection fibers: ALIC (anterior limb of the internal capsule) and PLIC (posterior limb of the internal capsule); **(B)** The association fibers: CgC (superior cingulum) and UNC (uncinate fasciculus); **(C)** The commissural fibers: sCC (splenium of corpus callosum), bCC (body of corpus callosum) and gCC (genu of corpus callosum). Group effect *p* value was calculated using the linear mixed model.

**Supplementary Figure S6.**

**
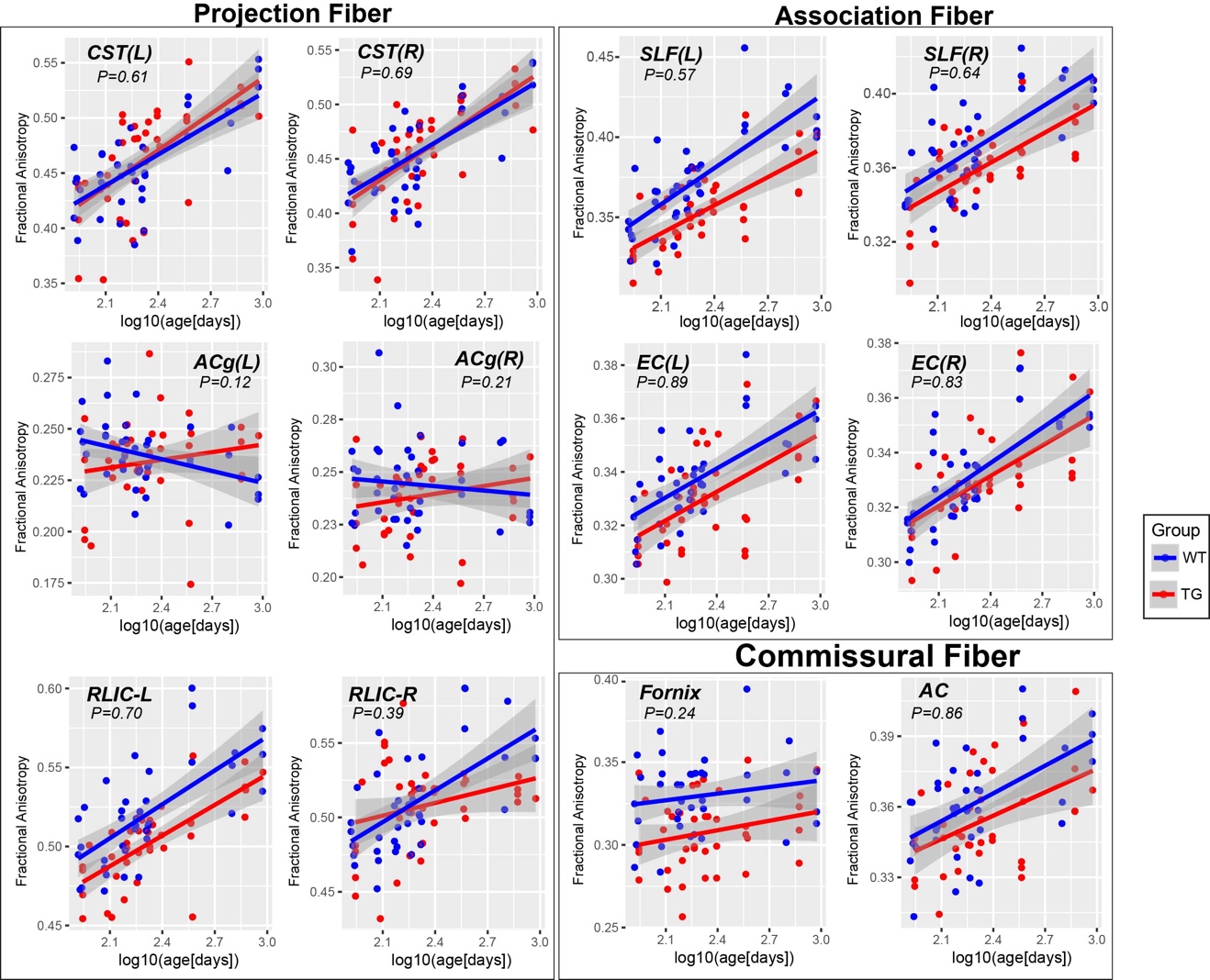
**

**Figure S6. The FA (fractional anisotropy) value change patterns of WM fibers during brain development**. The fibers include the projection fibers: CST (Corticospinal Tract) and ACg (Anterior Cingulum) and RLIC (Retrolenticular Limb of the Internal Capsule); the association fibers: SLF (Superior Longitudinal Fasciculus) and EC (External Capsule); the commissural fibers: Fornix and AC (Anterior Commissure). Group effect *p* value was calculated using the linear mixed model, and *p*<0.05 was taken as significant.

**Supplementary Figure S7.**

**
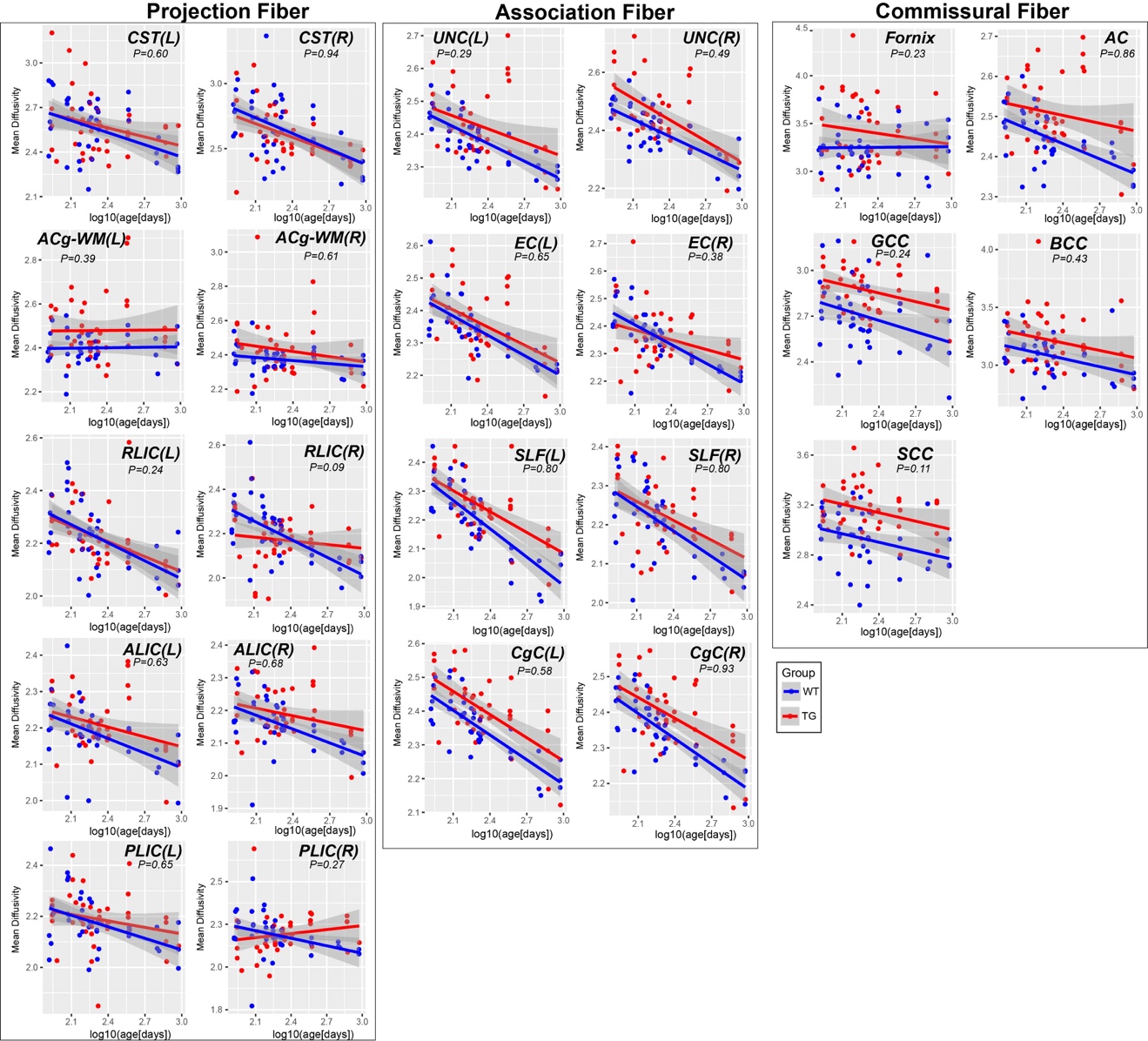
**

**Figure S7. The MD (mean diffusivity) value change patterns of the WM fibers during brain development**. The fibers include the projection fibers: CST (Corticospinal Tract), ACg (Anterior Cingulum), RLIC (Retrolenticular Limb of the Internal Capsule), ALIC (anterior limb of the internal capsule) and PLIC (Posterior Limb of the Internal Capsule); the association fibers: UNC (uncinate fasciculus), EC (External Capsule), CgC (Superior Cingulum) and SLF (Superior Longitudinal Fasciculus); the commissural fibers: Fornix, AC (Anterior Commissure), sCC (splenium of corpus callosum), bCC (body of corpus callosum) and gCC (genu of corpus callosum). Group effect *p* value was calculated using the linear mixed model, and *p*<0.05 was taken as significant.

**Supplementary Figure S8.**

**
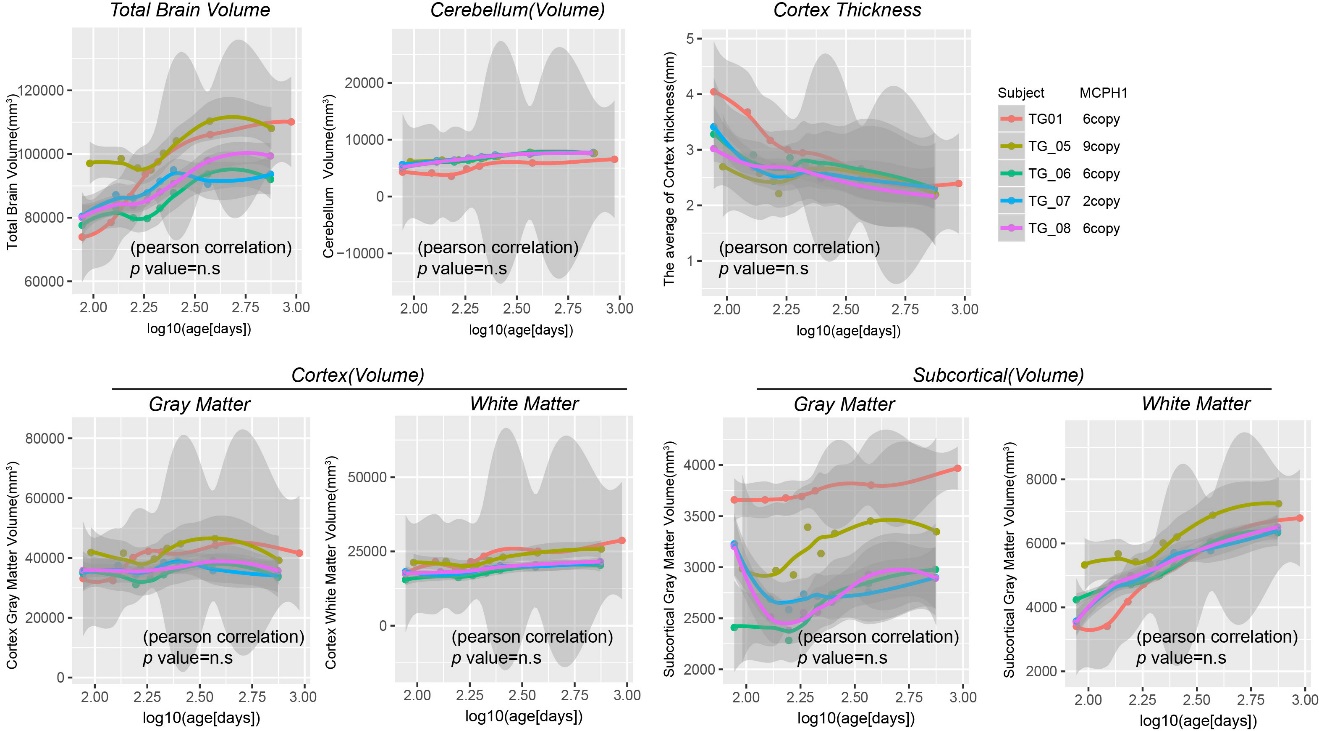
**

**Figure S8. Volume changes of brain regions of the TG monkeys carrying different huMCPH1 copies during development.**

**Supplementary Figure S9.**

**
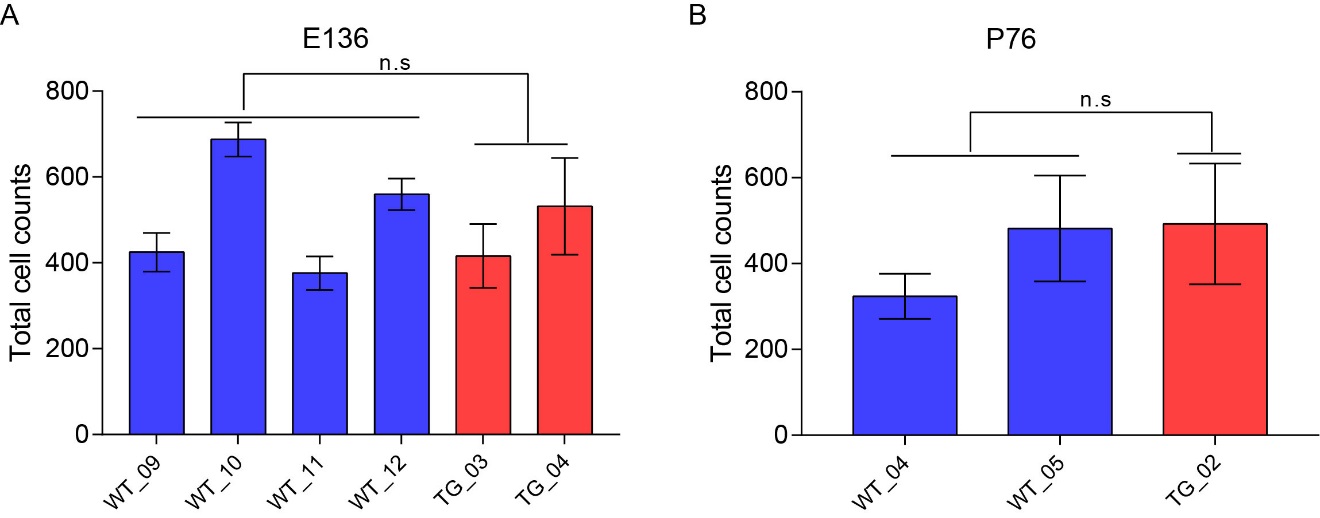
**

**Figure S9. Total cell number counts at two developmental stages (E136 and P76). (A-B)** Comparison of total cell numbers between the WT and TG monkeys at E136 and P76, and no difference was observed. The total cell number was calculated by counting the stained nucleus. The unpaired two tailed *t* test was used; n.s, not significant.

**Supplementary Figure S10.**

**
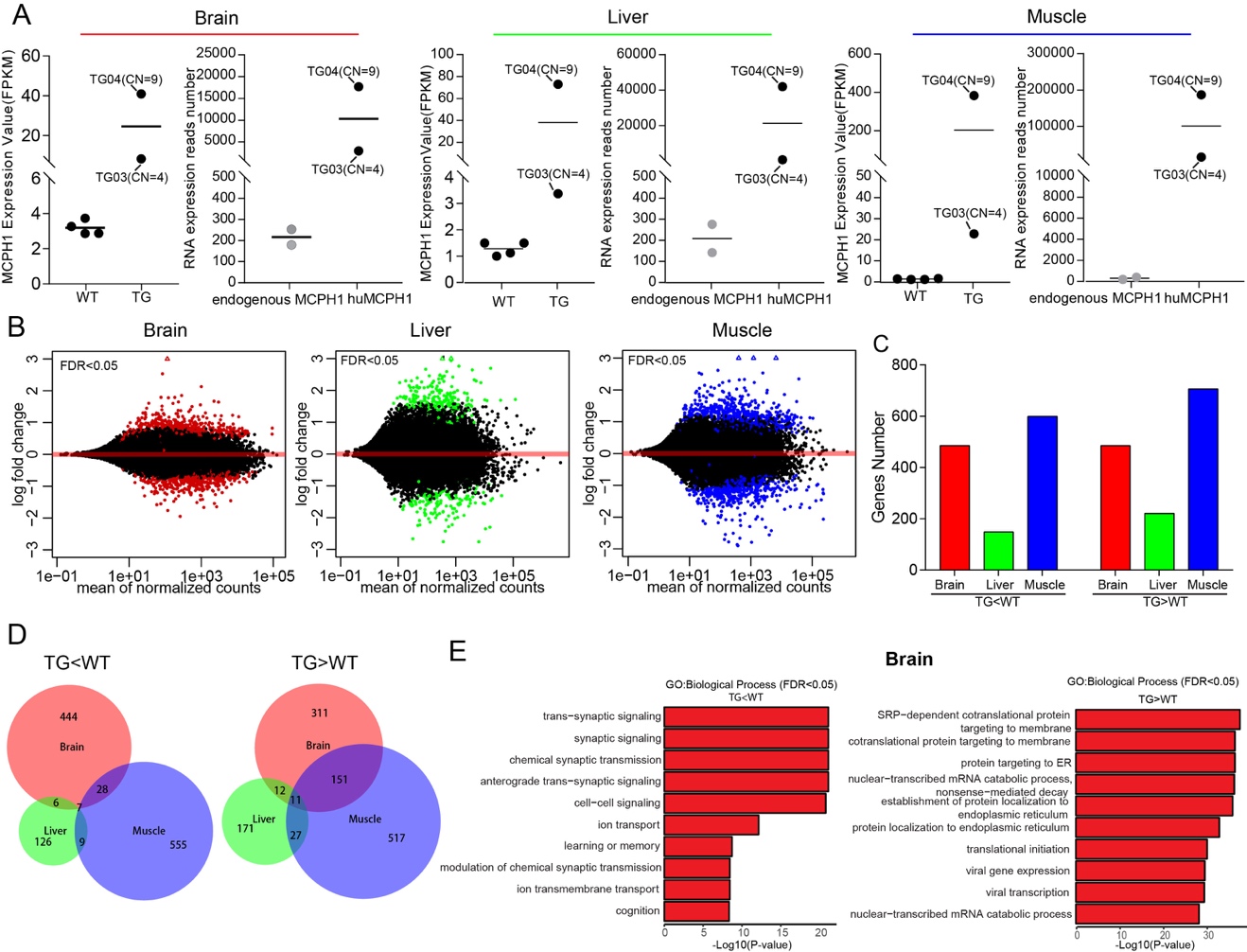
**

**Figure S10. Brain transcriptome analysis at E136. (A)** Every panel, Left plot: Comparison of expression levels of the transgene huMCPH1 and between the TG and WT monkeys. Right plot: comparison of expression levels between the transgene huMCPH1 and the endogenous monkey MCPH1 in the TG monkeys. CN stands for copy number. **(B)** MA plots of DEGs (differentially expressed genes between the TG and WT monkeys) in brain, liver and muscle. **(C)** The bar plots of DEG numbers in brain, liver and muscle. **(D)** Venn diagrams showing overlaps of DEGs among brain, liver and muscle. **(E)** The enriched gene clusters of DEGs in brain. The left panel indicates the down-regulated gene clusters, and the right panel indicates the up-regulated gene clusters.

**Supplementary Figure S11.**

**
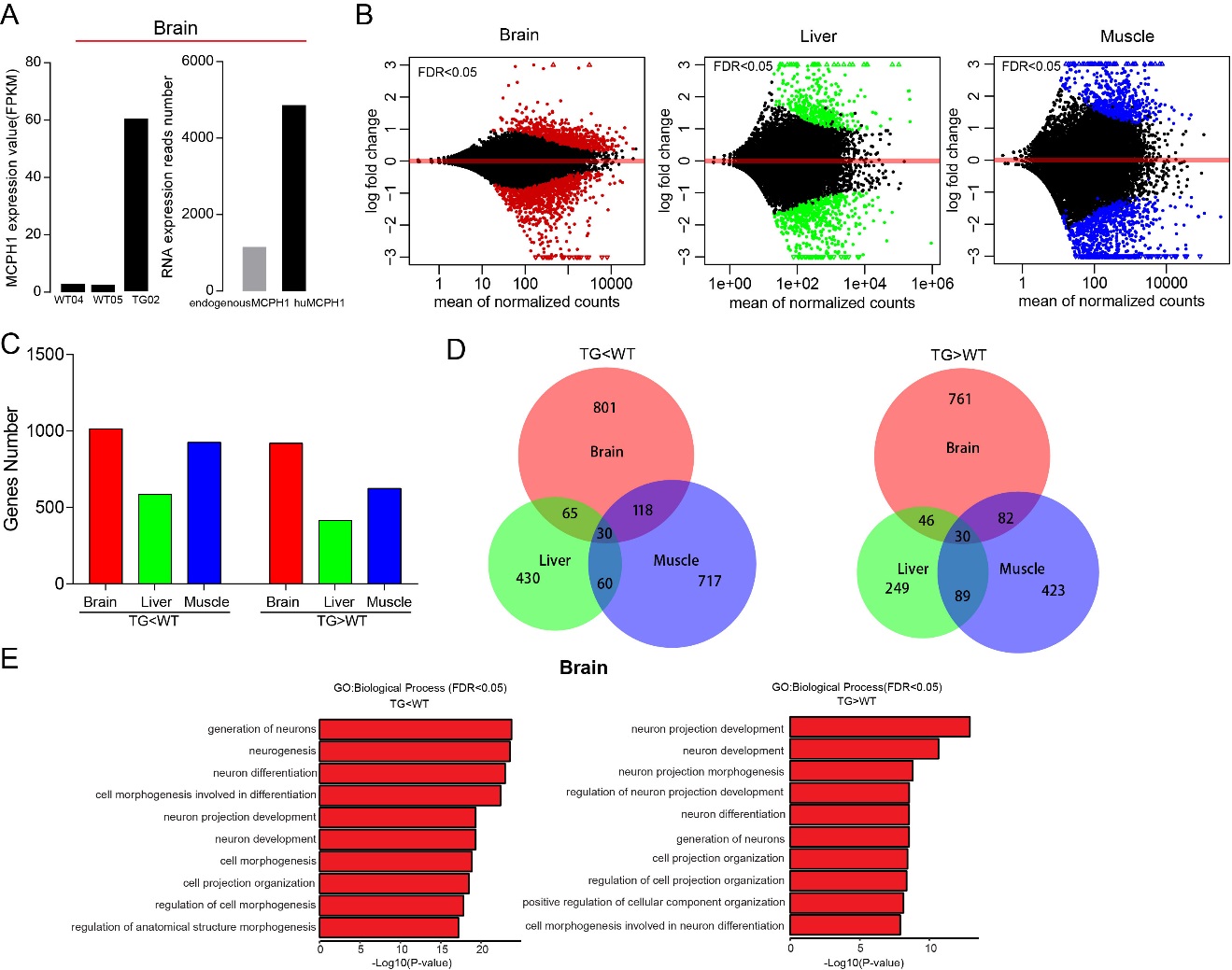
**

**Figure S11. Brain transcriptome analysis at P76. (A)** Left panel: comparison of brain expression levels of the transgene huMCPH1 between the TG and WT monkeys. Right panel: comparison of brain expression levels between the transgene huMCPH1 and the endogenous monkey MCPH1 in the TG monkey (TG-02). **(B)** MA plots of DEGs (differentially expressed genes between the TG and WT monkeys) in brain, liver and muscle. **(C)** The bar plots of DEG numbers in brain, liver and muscle. **(D)** Venn diagrams showing overlaps of DEGs among brain, liver and muscle. **(E)** The enriched gene clusters of DEGs in brain. The left panel indicates the down-regulated gene clusters, and the right panel indicates the up-regulated gene clusters.

**Supplementary Figure S12.**

**
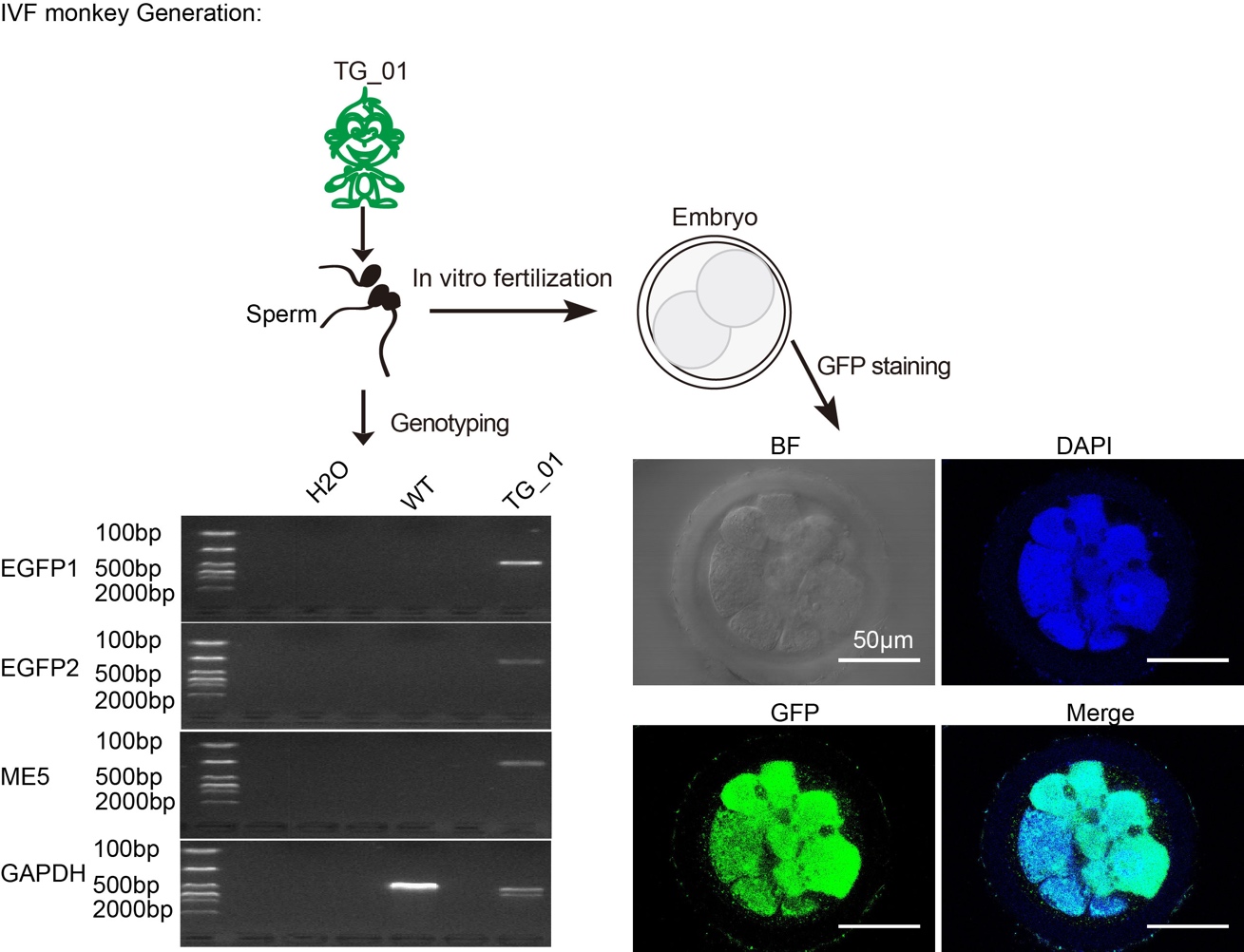
**

**Figure S12. Test of germline transmission of huMCPH1 in the male transgenic monkey (TG_01).** The left panel indicates the presence (by PCR amplification) of the transgene huMCPH1 in the sperms of TG_01. The house-keeping gene GAPDH was used as the internal control; The right panel shows the presence of eGFP fluorescent signals (green) in the monkey embryo fertilized with sperms from TG_01.

**Supplementary Figure S13.**

**
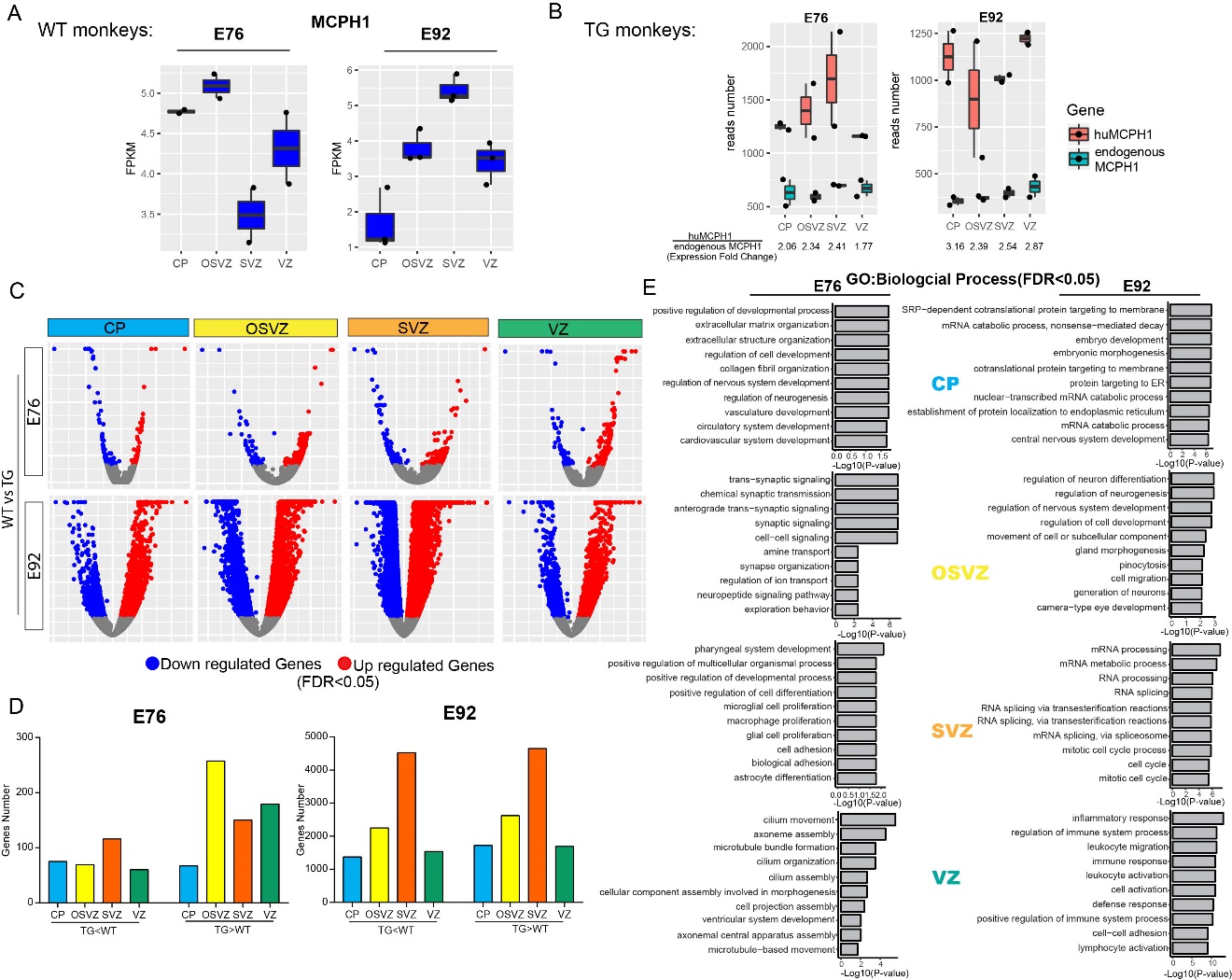
**

**Figure S13. Layer transcriptome comparison analysis between TG and WT at E76 and E92. (A)** MCPH1 expression pattern in of WT monkeys at E76 and E92. **(B)** Comparison of layer expression levels between the transgene huMCPH1 and the endogenous monkey MCPH1 in the E76 and E92 TG monkeys. **(C)** Volcano plots of differentially expressed genes between the TG and the WT monkeys at the indicated cortical laminae. **(D)** The bar plots of DEG numbers in CP, OSVZ, SVZ and VZ layers at E76 and E92. **(E)** Enriched gene clusters of differentially expressed genes (TG vs. WT) in CP, OSVZ, SVZ, and VZ laminae at E76 and E92**.**

**Supplementary Figure S14.**

**
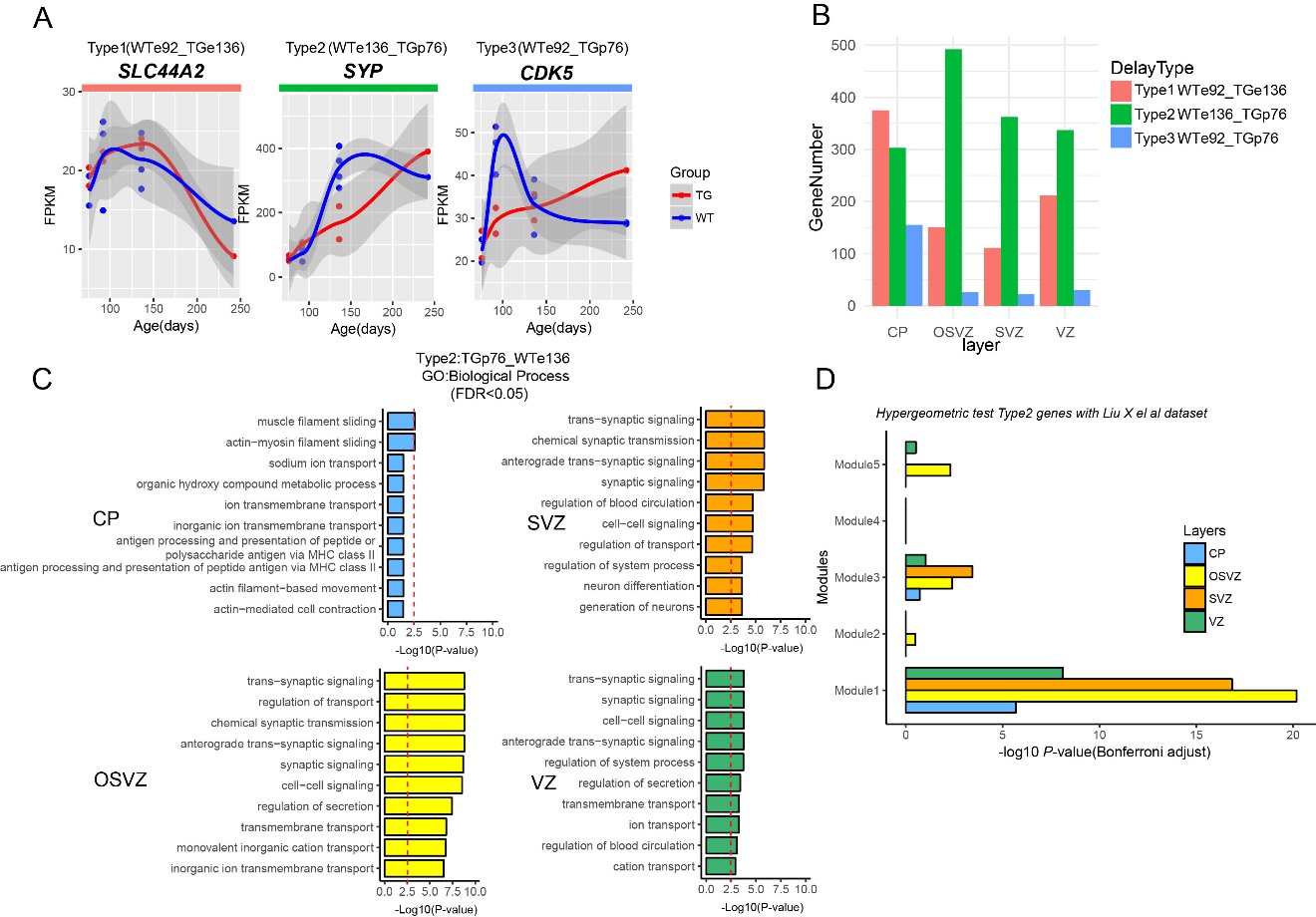
**

**Figure S14. Time series transcriptome analysis reveals gene expression delay in the TG monkeys. (A)** Gene case examples of three types of gene expression delay. **(B)** Bar plots of gene number counts of three types of differential gene expression patterns in different cortical laminae. **(C)** GO ontology analysis of the Type-2 genes showing enrichment of synapse functions. **(D)** The Type-2 genes are significantly overlapped with the reported Module-1 human-specific delay genes. The hypergeometric test was used.

**Supplementary Figure S15.**

**
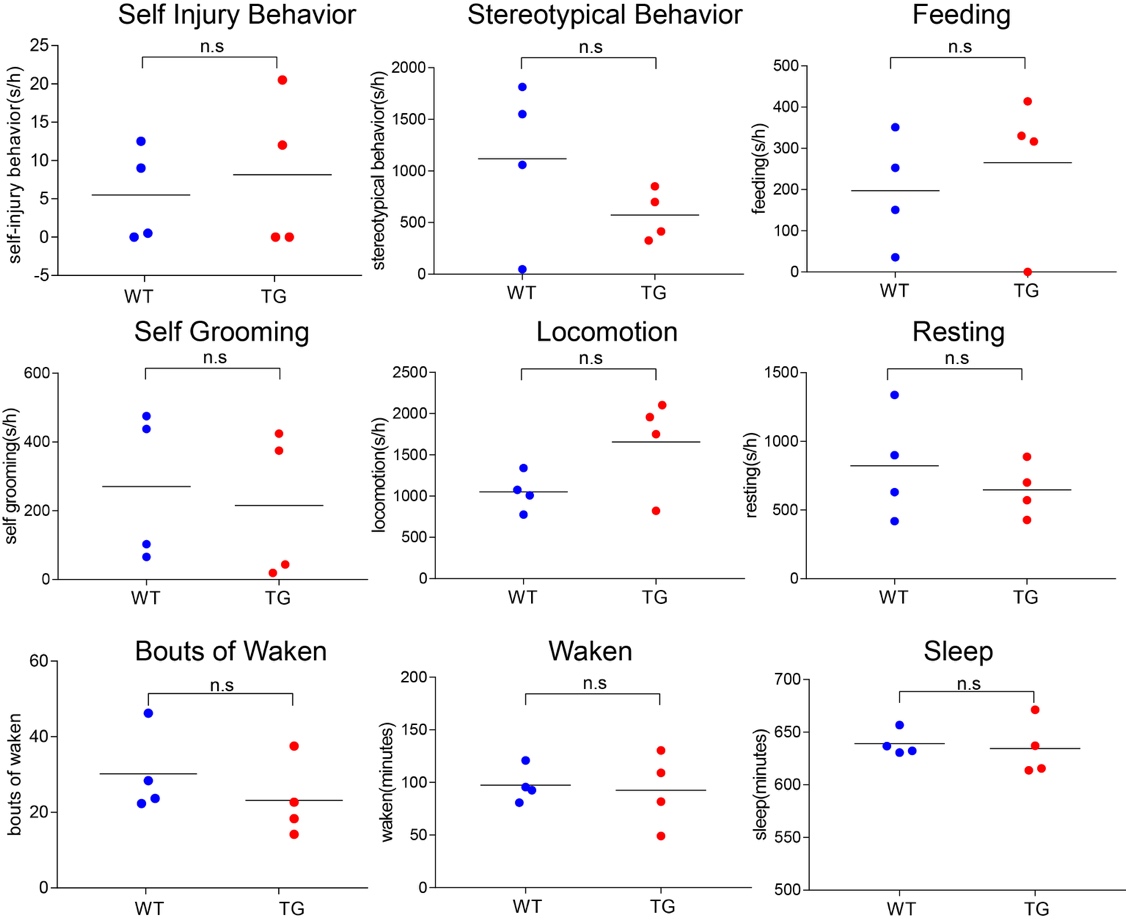
**

**Figure S15. Results of general behavior analysis.** The behavioral data included 4 days’ recordings of the monkeys. The Mann-Whitney U test was used for self-injuring behavior, and the Student’s t-test was used for the other behaviors.

**Supplementary Figure S16.**

**
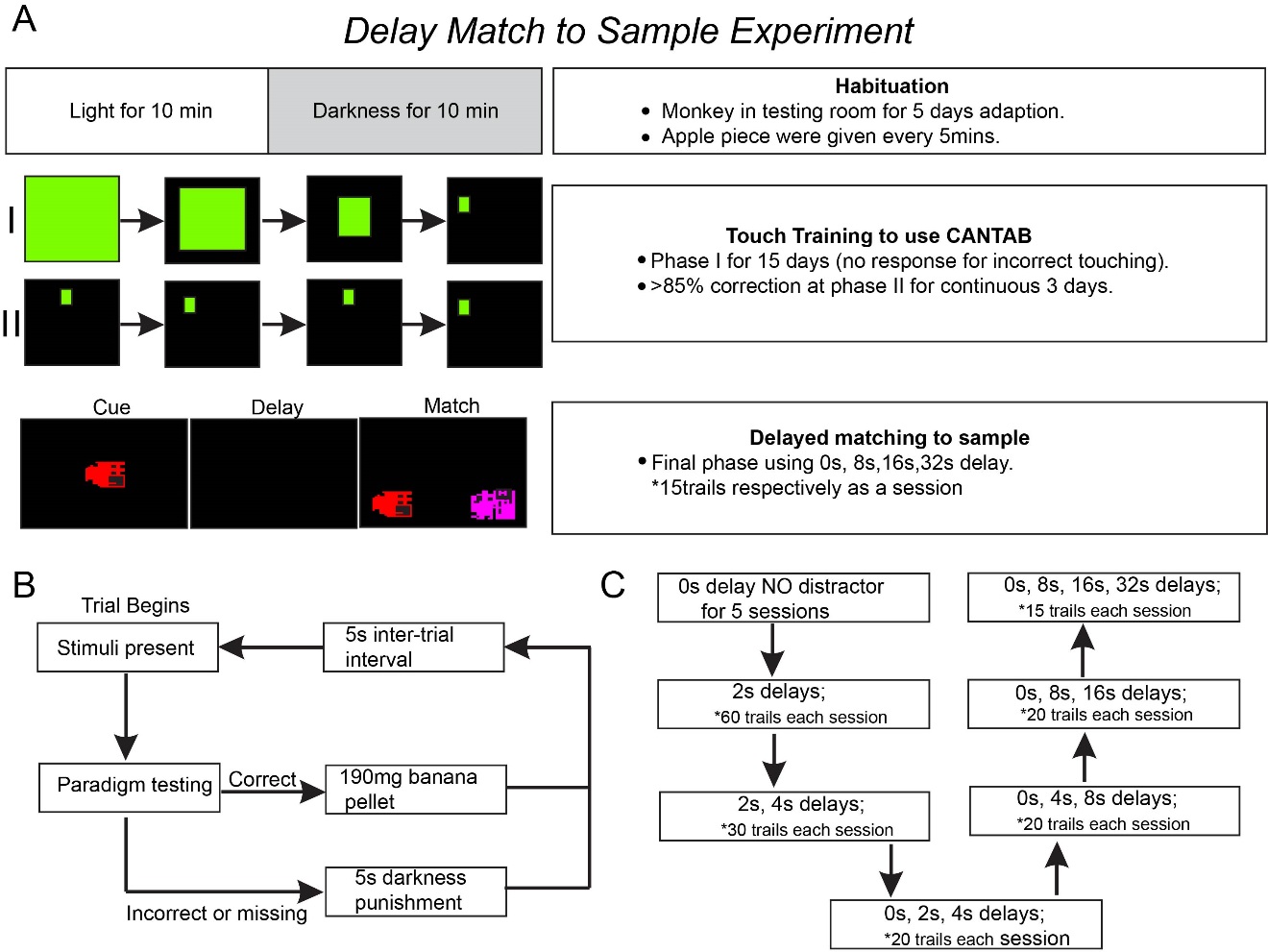
**

**Figure S16. The pipeline of DMS (Delay Match to Sample) experiments.**
