# Supplementary material for: Transgenic rhesus monkeys carrying the human MCPH1 gene copies show human-like neoteny of brain development
Source: Natl Sci Rev. 2019 Mar 27;6(3):480–93. doi: 10.1093/nsr/nwz043 (PMC8291473; doi:10.1093/nsr/nwz043)
Supplement: nwz043_Supplemental_Files [file nwz043_supplemental_files.zip › SupplementaryData_Methods_final.docx]

**Methods**

***Animal subjects***

Totally, 21 wild type monkeys (3 monkey feeding monkeys, 5 human feeding monkeys and 13 embryonic monkeys) (Table S2) and 11 transgenic monkeys (6 human feeding TG monkeys and 5 embryonic TG monkeys) (Table1) were analyzed in this study. All monkeys were from Yunnan Key Laboratory of Primate Biomedical Research. All animal procedures were conducted following the international standards, and were approved in advance by the Institutional Animal Care and Use Committee of Kunming Institute of Zoology, Chinese Academy of Sciences and Yunnan Key Laboratory of Primate Biomedical Research (Approval No: SYDW-2010002 and KBI_K001115033-01,01).

***Cell culture***

The HEK293T cell line was obtained from ATCC and cultured in Dulbecco Modified Eagle Medium (Gibco, Rockville, MD) with 10% fetal bovine serum (HyClone, Logan, UT) at 37℃ in a humidified atmosphere containing 5% CO_2_.

***Targeting expression vector cloning***

The full-length coding region of human MCPH1 was PCR amplified, and the PCR products were digested with Age I and EcoR I before being cloned into a frame for N-terminal fusions into the simian immunodeficiency virus (SIV) based vectors, GAE-CAG-huMCPH1-eGFP/WPRE, which harbors the sequence encoding eGFP, GAE-CAG-EGFP expression plasmid [1]. The final constructs were confirmed by sequencing on an ABI-3130 Automatic Sequencer.

***Transient transfection***

All transfections were carried out in triplicates using 24-well plates (Corning, NY, USA). About 2×10^5^ cells were seeded for 24hrs prior to transfection. Briefly, the equal numbers of cells were plated in 24-well and 6-well plates and were grown to 80% confluence. The indicated amounts of vectors were mixed in OPTI-MEM medium (Gibco) with lipofectamine2000 (Invitrogen) and the solution was incubated for 30 min at room temperature, and then placed on the cultured cells. After 6 hr, the medium was changed, and Dulbecco Modified Eagle Medium (Gibco) with 10% fetal bovine serum (Hyclone) was used.

***Virus production and gene delivery in rhesus monkey pre-implantation embryos***

The amount of 2.5 × 10^6^ 293T cells were seeded in a dish 10cm in diameter 24 hrs before transfection. Cells were transfected by the lipo-2000 method in a mixture of DNAs containing 10ug *pSIV3+* plasmid encoding gag, pol, tat and rev proteins, 13ug simian immunodeficiency virus (SIV)-based vectors, GAE-CAG-tgMCPH1-eGFP/WPRE, which harbors the sequence encoding humanMCPH1-eGFP, 10ug *pGRev* plasmid encoding the vesicular stomatitis virus glycoprotein(VSV-G). In the next day, cells were fed again with fresh medium and further cultured for 36-48 hours. The supernatant then was clarified by centrifugation (3,000g, 15 min), passed through a cellulose acetate filter (pore size, 0.8um), and concentrated by ultracentrifugation (25,000 RPM, 2h) on a 20% (wt/vol) sucrose gradient. The viral pellet was resuspended in PBS, frozen, and titrated by infection of 293T cells followed by counting of EGFP-positive cells. Early-cleavage-stage embryos were selected for perivitelline space injection. A lentiviral suspension was loaded into the injection needle by micropipette before injection into the perivitelline space. After virus injection, the embryos were cultured in HECM-10 before transfer into surrogate mothers.

***Collection of rhesus monkey oocytes and in vitro fertilization***

Cycling females (6 to 12 year old) were subjected to follicular stimulation using twice-daily intramuscular injections of 18 IU of recombinant human FSH(rhFSH) (Gonal FTM Laboratories) for 8d; then 1,000 IU of human chorionic gonadotropin(hCG) (Lizhu Groups) were injected on day 9 as described by Niu et al [2]. Cumulus-oocyte complexes were collected from animals by laparoscopic follicular aspiration 30-34 h following hCG administration. Follicular contents were placed in Hepes-buffered Tyrode’s albumin lactate pyruvate (TALP) medium containing 0.3%BSA at 37℃. Oocytes were stripped of cumulus cells by pipetting after brief exposure (<1min) to hyaluronidase(0.5mg/ml) in TALP-Hepes to allow visual classification of nuclear maturity as prophase I (PI; intact germinal vesicle), metaphase (MI; no germinal vesicle, no polar body), metaphaseⅡ(MⅡ; first polar body present), and atretic (presence of fragmentation or vacuoles in ooplasm). Immature oocytes in either MI or PI stages were cultured in a 50 µl drop of CMRL-1066 medium (Invitrogen) containing 10% FBS, 10 IU/ml porcine FSH, and 10 IU/ml ovine luteinizing hormone at 37℃ in humidified air (5% CO2) for up to 24h. Oocytes that were mature (MⅡ) at collection were placed in chemically defined, protein-free hamster embryo culture medium-10 (HECM-10) at 37℃ in 5% CO2 until inseminated (36 h) with capacitated, hyperactivated spermatozoa diluted to a final concentration of 2 × 105/ml in 50-ul drops of TALP for fertilization. After coincubation of oocytes and spermatozoa for 12-16 h, oocytes were examined for the presence of two pronuclei and two polar bodies as evidence of fertilization. Fertilized ova were washed to remove spermatozoa and then cultured in HECM-10 containing 10% FCS (HyClone, Logan, UT) to allow embryo development. Culture medium was replaced every other day.

***Selection of surrogate mothers and embryo transfer***

Embryos at the 4-cell to blastocyst stage were selected for embryo transfer. Surrogate females exhibiting normal menstrual cycles were identified based on their steroid hormone profiles and observation of menses. Embryo transfer into the oviduct was conducted by the laparoscopic approach. In brief, monkeys were anesthetized with ketamine (10-12mg/kg). After sterile skin preparation and draping, the abdomen was insufflated with CO_2_ at 15 mm Hg pressure, and the endoscopes was inserted through the corresponding trocar cannula via a small supraumbilical incision. Typically, three or four embryos were transferred unilaterally into each female using a polythene catheter connected to a 1-ml syringe filled with TH3 medium. The catheter containing the embryos was inserted transabdominally by threading it through a 25-gauge hypodermic needle and was advanced through the fimbrium into the oviductal ampulla to a distance of about 3cm, where the embryos were released. After transfer, the catheter was removed and carefully examined before being rinsed to ensure that all embryos had been expelled. In the event an embryo was retained, a second transfer was attempted. To detect pregnancy, serum estradiol and progesterone concentrations were monitored, and after initial signs clinical pregnancy was confirmed by fetal cardiac activity detected via ultrasonography. The progression of pregnancies was monitored periodically by ultrasonography.

***Genotyping***

To detect the MCPH1-EGFP fusion gene, two pair GFP specific primers eGFP1 F:5CGTCCAGGAGCGCACCATCTTC3, R:5GGTCTTTGCTCAGGGCGGACT3; eGFP2 F:5GACTTCTTCAAGTCCGCCAT3, R:5ACCTTGATGCCGTTCTTCTG3; and three pair MCPH1-GFP primers were used to detect after amplification of genomic DNA from monkey tissues including placenta, umbilical cord, liver, brain and muscle.

***Expression analysis***

To investigate the mRNA expression of transgenic monkeys, we analyzed human MCPH1 expression in the monkey placenta tissue. RT-PCR procedure is as follows: Total RNA was extracted using TRIzol (Invitrogen, Carlsbad ,CA). The RNA was treated with DNase (Fermentas) to remove possible genomic DNA contamination, then subject to reverse transcription using oligo-dT(20) primer and Ominicript Reverse transcriptase (Qiagen, Valencia, CA). RT-PCR primers as follows: eGFP3 F:5TGACCTACGGCGTGCAGTGCTT3,R:5TCGTCCATGCCGAGAGTGATCC3;eGFP45AACGTCTATATCATGGCCGACAAGCA3,5CTCGTCCATGCCGAGAGTGATCC3; ME5F: 5CACCCAGCACAAGGTCTGTGC3; ME5R: 5GGACACGCTGAACTTGTGGCC3;ME1F:5GGCTTTTCTTACACCATTGAGGACC3;ME2F:5TACCCGGAGGATACAGTGGAAGTGT3;ME3F:5ATTGGGTCACTGGATTTCTGAGGAG3; ME4F: 5CATCGGGCCCTACAGCGGA3;

***Western blot***

Proteins from the monkey placenta, brain, kidney and liver were homogenized in RIPA lysis buffer (50mM Tris-HCl,pH 7.4;150mM NaCl;1mM EDTA;1% Triton-100;1mM Na_3_VO_4_) containing a cocktail of protease inhibitor (Sigma Chemical, St. Louis, MO). Extracted proteins (15-20μg) were separated by SDS-polyacrylamide gel electrophoresis and transferred to a membrane incubated with anti-actin monoclonal antibody (Abcam, Cambride, MA) and anti-MCPH1 monoclonal antibody (Abcam, Cambride, MA) separately. Immunoreactivity was detected with an enhanced chemiluminescence system (Pierce Protein, Rockford, IL) with colored markers (Fermentas, Lithuania) as the molecular size standard.

***Determination of huMCPH1 integration sites using captured sequencing***

The reported method was employed to determine the huMCPH1 integration sites in the TG monkeys [3]. In brief, genomic DNA extracted from monkey tissues was used to construct a DNA library. Sequencing linkers were further added onto genomic segments (length around 500–700 base pairs (bp)). After end repairing and 3’ A-adding, the fragmented DNAs were ligated with Y-shape adaptor. Amplification was performed with the adaptor primers. Asymmetry-primer PCR (APP) was used to enrich the viral integration sites in each library. The APP method includes two PCR systems. The first PCR system includes only LTR specific primer. After 12 cycles of linear amplification, adaptor specific primer was added in the PCR system followed by 12 cycles of exponential amplification. PCR products were purified using 0.7 × AMPure beads (Beckman, A63882). The second PCR system uses a pair of primers nest the primers in the first PCR system. After 12 cycles of linear amplification and 15 cycles of exponential amplification, the PCR products of 500–700 bp in size were isolated by agarose gel electrophoresis before being used to construct libraries with Illumina paired-end adapters according to the manufacturer protocol and sequenced by Illumina MiSeq V3 (2×300 base paired ends). SummonChimera software [4] was used to infer SIV virus integration sites and only the paired-end reads showing the fusions of viral sequences and the macaque genome segments were selected. The reads showing the same integration position were merged and treated as a unique integration site. The integration sites identified have to satisfy the below conditions:1) at least two chimeric reads at different position supporting the same integration sites; 2) The integration sites are shared in the two tissues of the same transgenic monkey or by cloning confirmation; Target sequences containing LTR of transgene cassettes and genomic segments flanking the transgenes were analyzed. Primer sequences are:

SIV-LTR1F: 5GACCTCTTCAATAAAGCTGCCATTT3;

Adapter_1R: 5GGATAGCGACGCACGGAACTCT3;

SIV-LTR2F: 5TCTGTTAGGACCCTTTCTGCTTT3;

Adapter _2R: 5CTCCATCTCATCCTGCGTGTC3;

***In vitro immunofluorescence staining of cells***

Isolated umbilical cord endothelial cells or MEF cells were washed several times in PBS before being fixed in in 3.70% paraformaldehyde for 15-20 min and washed again twice in PBS+0.3%PVP (polyvinylpyrrolidone); Permeabilize samples in 0.25% Triton X-100 for 30 min and wash in PBS (3 x 5 mins). Sample were blocked in 1% BSA in PBS for at least 1 hour at room temperature, and then incubated with primary antibodies anti-GFP(Invitrogen) (diluted in 1%BSA/PBS, blocking solution) for 1 hour at room temperature (usually the primary antibody incubation is done at 4°C overnight), wash three times in PBS+0.1% Tween-20 (3 x 5mins). The samples were incubated with second antibody (1:200 in 1%BSA/PBS) protected from light for 1 hour at room temperature and then washed three times in PBS+0.1% tween-20 (3 x 5mins). Nuclei were stained with DAPI (10μg/ml), and then washed several times in PBS.

***MRI acquisition***

All monkeys were scanned on a Philips MRI machine (Philips Achieva 3.0T TX) at the Kunming Medical School Image Center. Ketamine hydrochloride (10mg/kg) followed by pentobarbital sodium (10mg/kg) was used for immobilization during scanning. T1-weighted images were acquired using a 3D turbo field echo sequence (time repetition (TR) = 14ms, time echo (TE)=7ms, flip angle (FA) = 8°, field of view (FOV) = 120mm x120mm x 55mm, acquisition matrix size = 240 ×187 x 55, acquisition voxel size = 0.5 x 0.64 x 1 mm3, reconstruction voxel size = 0.375 x 0.375 x 0.5 mm3, number of average (NSA) = 3, scan duration = 10 min). Diffusion weighted images (DWIs) were acquired using a 2D echo-planar spin-echo sequence (TR = 5348ms; TE = 88ms; FA = 90°; FOV = 120 x 120mm; acquisition matrix size = 100 x 100; slice thickness = 1.2mm, no gap, acquisition voxel size = 1.2 x 1.2 x 1.2 mm3, reconstruction voxel size = 0.75 x 0.75 x 1.2 mm3, NSA = 4, b = 800 s/mm2, 32 directions with one b0 image, scan duration = 13 min).

***T1w data preprocessing***

Brain extraction was performed using a semi-automatic atlas-fusion method: the infant macaque atlas (with a brain mask) [5] were registered and fused to each monkey to generate an initial brain mask, which was further corrected manually (Fig.S2A). The manually corrected mask was then post-processed using an automatic approach to reduce the human bias from manual correction, which was described in detailed in the later section (Fig.S2E). FAST in FSL [6, 7] was used to remove bias filed and segment each T1w image into partial volume images of the gray matter, the white matter, and the cerebrospinal fluid. Meanwhile, cortical thickness was also estimated used the DiReCT [8], method implemented in the Advanced Normalization Tools (ANTs) [9]. All images were registered to the study-specific T1w template using the ANTs as described in the later section. To facilitate ROI-based analyses, the brain parcellation of the infant macaque atlas [5] was transformed to the T1w template, fused into the native space of each scan and used for the analyses of tissue volumes and cortical thickness.

***DTI data preprocessing***

Eddy currents and motion data were corrected using the Diffusion Toolbox of FSL [10], and brain extraction was performed using a semi-automatic template-based method as mentioned above (Fig.S2B). Diffusion tensor-based measurements were then calculated, including fractional anisotropy (FA), mean diffusivity (MD), axial diffusivity (AD) and radial diffusivity (RD). All tensor images were registered to the DTI template as described in the next section using DTI-TK, which was a spatial normalization tool optimized for white matter morphometry [11]. The DTI-TK utilized higher order information of diffusion tensor images for spatial normalization and had been used to generate high quality DTI templates of adult humans [12], human infants [13], adult macaques [14] and infant macaques [5]. To facilitate analyses of white matter properties, the UW-DTIRMAC271 white matter atlas [15] was transformed to the DTI template, fused into each scan. To ensure ROIs were located at white matter regions, each ROI was first dilated by one voxel and then masked by an FA mask (FA>0.2**)** in each scan. Finally, diffusion tensor-based measurements (FA, MD, AD, and RD) were averaged for each white matter ROI.

***MRI image template construction***

Two different approaches were tested for constructing the T1w and DTI template, both of which were aimed to avoid bias to any particular time point or monkey (Fig.S2A-C). For the first approach, the final templates were constructed directly based on all scans from all monkeys. For the second approach, animal-specific templates were first created using all scans from each monkey and then these animal-specific templates were used to generate the final templates; the transforms from the two steps were concatenated so that the input images were only re-sampled once to minimize interpolation errors. All templates were created via an iterative procedure, in which rigid alignments, affine transformation, and nonlinear transformation were repeated until the average image converged for each transformation (Fig.S2D). The brain parcellation of the infant macaque atlas [5] and the white matter ROIs of the UW-DTIRMAC271 atlas [15] were also transformed to the final T1w and the final DTI template respectively. These brain parcellations and white matter ROIs were fused into the native space of each scan for the ROI-based analyses. The final templates from the two approaches were highly similar. As all analyses were performed in the native space, there was no difference between the results from the two approaches. The final results reported in the study was based on the second approach.

***Brain mask bias reduction using automatic approach***

The above brain extraction methods involved manual correction, which may introduce human bias on different scans. To correct the potential bias, brain masks were dilated by 10 voxels to include parts of the brain skull. A new template with brain skull was then created using the second template construction method. Brain masks from each scan were transformed into the template and averaged to create one brain probability mask. The probability mask was binarized (by a threshold of 0.5), manual corrected and nonlinearly transformed back to the native space of each scan for the final brain extraction.

***Probability maps (priors) guided tissue segmentation***

As the white matter of the infant monkeys were not fully myelinated, the T1w may not have enough gray and white matter contrast for an accurate segmentation using the default k-means algorithm of FAST/FSL to initiate the segment without priors. To facilitate the segmentation, tissue probability maps were created from the original segmentation of later time-points (>16-month) using the template construction approach. The probability maps were then nonlinearly transformed into each scan. The T1w image of each scan was re-segmented by using the probability maps as priors, which effectively improve the segmentation of infant monkey brain (Fig.S2F).

***Brain tissue section analysis***

Monkey frontal cortex tissues were fixed in 4% paraformaldehyde overnight, transferred to 30% sucrose, stored at 4°C, embedded and then cut into 8 µm sections. For detecting GFAP, NeuN, DCX and FABP, sections were deparaffined with xylene and then moved from alcohols to water; Washing with water followed in PBS for 5min; Antigen retrieval with citrate pH6 solution at 90~100℃ for 5min, cool down for 30 min at RT, rinse in 3minPBS at RT. Follow in 3%H_2_O_2_for 10 min; Rinse in PBS 3min at RT, Incubate tissue in Biotin Blocking System for 20min at RT; GFAP antibody (Gene Tec, Shanghai, China, dilution 1:100), NeuN antibody (Millpore, MA, USA, dilution 1:100), DCX antibody (Novus, CO, USA, dilution 1:200) and FABP antibody (Novus, CO, USA, dilution 1:1000), for 1h at RT separately, Rinse in PBS for 3min at RT, Second antibody(dilution 1:500) for 30min at RT, Rinse in PBS for 3min; Incubate sections in DAB solution for 5min, Rinse slides with water for 5min; Hamatoxylin for 2s, Rinse slides with flow water for 2min; Dehydrate in gradient concentration alcohol and clear in xylene; Mount with resinous mounting medium. Cell counting was performed using a double-blinded method, and each monkey had 3 or 4 staining slices and we counted 4 visual fields per slice.

***RNA isolation***

Within 24 hours after monkeys died, we dissected the monkeys and put tissues into liquid nitrogen to prevent RNA degradation. When we extract RNA, tissues were homogenized in TRIzol (Invitrogen), and RNA extracted by chloroform extraction and purified using Qiagen RNEasy colums. All RNA quality checks were performed as following: firstly, we run RNA gel to check RNA; Secondly, using Qubit fluorometer to measure concentration of RNA; thirdly, using Agilent 2100 to check RNA integrity; Finally, using Nanodrop to check RNA purity. The RIN value was used to measure RNA integrity. Nearly all RNA samples had a RIN value over 7.0.

***Laser microdissection***

The fetal monkey frontal cortex tissues were partitioned into several sections, after that, the specimens were embedded in OCT and then gradually frozen in an ethnol /dry ice, and stored at −80°C until processing. Frozen tissue was cryosectioned at 30μm onto polyethylene naphthalate (PEN) slides (Leica Microsystems) and place immediately into microslide box on dry ice, and stored at -80°C until use.

Taking the section slides from the -80°C freezer and put them on a blotting paper for 30s, and then transfer the slides into the 95% EtOH for 30s, 75% EtOH for 30s and 50% EtOH for 30s, place the slides on a blotting paper. Add 400ul Nissl stain solution on the slide, and incubate at room temperature for 40s. Proceed through the dehydration series (50%, 75% and 95% EtOH) till the two rounds of 100% EtOH, and leave the slides in the final xylene jar for 5 min. After air-drying in the fume hood for 5 min, proceed immediately with laser capture microdissection (LCM).

Laser microdissection was performed on a Leica LMD6000 (Leica Microsystems). Microdissected tissues including CP, OSVZ, SVZ and VZ were collected directly into QIAzol buffer from the miRNeasy Mini Kit (Qiagen) in a 0.2ml tubes, vortexed, centrifuged, and frozen at −80 °C. RNA was isolated following the manufacturer’s directions for the miRNeasy Mini Kit. RNA samples were run on the Agilent 2100 Bioanalyzer (Agilent Technologies).

***cDNA library preparation***

RNA-seq libraries were generated according to standard procedures using Illumina library preparation kits under the manufacturer’s protocols (Illumina, San Diego, USA). Briefly, for postnatal monkey tissues, after extracting the total RNA from the samples, mRNA is enriched by using the oligo(dT) magnetic beads and also mRNA is enriched just by removing rRNAs from the total RNA. Adding the fragmentation buffer, the mRNA is interrupted to short fragments (about 200 bp), then the first strand cDNA is synthesized by random hexamer-primer using the mRNA fragments as templates. Buffer, dNTPs, RNase H and DNA polymerase I are added to sythsize the second strand. The double strand cDNA is purified with QiaQuick PCR extraction kit and washed with EB buffer for end repair and single nucleotide A (adenine) addition. Finally, sequencing adaptors are ligated to the fragments. The required fragments were purified by agrose gel electrophoresis and enriched by PCR amplification. The Single end library products are ready for sequencing analysis via Illumina HiSeq™ 2000 (Illumina, San Diego, USA). For Prenatal monkey tissues, a total amount of 3 μg RNA per sample was used as input material for the RNA sample preparations. Sequencing libraries were generated using NEBNext® UltraTM Directional RNA Library Prep Kit for Illumina® (NEB, USA) following manufacturer’s recommendations and index codes were added to attribute sequences to each sample. mRNA was purified from total RNA using poly-T oligo-attached magnetic beads. Fragmentation was carried out using divalent cations under elevated temperature in NEBNext First Strand Synthesis Reaction Buffer (5X). First strand cDNA was synthesized using random hexamer primer and M-MuLV Reverse Transcriptase (RNaseH-). Second strand cDNA synthesis was subsequently performed using DNA polymerase I and RNase H. In the reaction buffer, dNTPs with dTTP were replaced by dUTP. Remaining overhangs were converted into blunt ends via exonuclease/polymerase activities. After adenylation of 3’ ends of DNA fragments, NEBNext Adaptor with hairpin loop structure were ligated to prepare for hybridization. In order to select cDNA fragments with right length, the library fragments were purified with AMPure XP system (Beckman Coulter, Beverly, USA). Then 3 μl USER Enzyme (NEB, USA) was used with size-selected, adaptor-ligated cDNA at 37°C for 15 min followed by 5 min at 95°C before PCR. Then PCR was performed with Phusion High-Fidelity DNA polymerase, Universal PCR primers and Index (X) Primer. At last, products were purified (AMPure XP system) and library quality was assessed on the Agilent Bioanalyzer 2100 system. The clustering of the index-coded samples was performed on a cBot Cluster Generation System using TruSeq PE Cluster Kit v3-cBot-HS (Illumina) according to the manufacturer’s instructions. After cluster generation, the library preparations were sequenced on an Illumina Hiseq 4000 platform and paired-end reads were generated. The RNAseq data have been deposited in the GEO database (GSE56962) and the GSA (Genome Sequence Archive) database (CRA000343).

***Bioinformatics analysis***

Illumina reads were processed by trimming adapter sequences, while remaining reads were mapped to the respective reference macaque genome using HISAT2 2.1.0 [16]. We quantified gene expression from the mapped reads using HTSeq-count (http://www-huber.embl.de/users/anders/HTSeq/doc/count.html) that obtained integer counts of mapped reads per gene. Cufflinks was used to obtain FPKM expression values, using automatic estimation of the library size distributions and sequence composition bias correction [17]. Differentially expressed genes were identified based on integer count data using DESeq2 [18] R package, which determines DE by modeling count data using a negative binomial distribution as follows: First, size factors are calculated that take into account the total number of reads in different samples. Second, a dispersion parameter is determined for each gene which accounts for biological variation between samples. Third, a negative binomial distribution is used to fit the counts for each gene. The *p*-value is calculated based on the wald test. The *p* values adjusted for multiple testing was calculated using the Benjamini-Hochberg procedure, which controls false discovery rate (FDR<0.05). For the list of differentially mRNA genes, we tested weather each had enriched GO terms in biological process and molecular functions using the ToppGene Suite [19]. Only those functional annotation terms associated with the various sets of differentially expressed genes were clustered that were significantly enriched (Benjamini and Hochberg correction, *p*<0.05) compared with the function annotation terms associated with the total population of genes expressed in at least one of the transgenic or control samples.

In order to identify delay genes in the TG monkeys, according to wild type monkeys genes expression peak time, we divided the delay gene types into three types: Type-1: WTe92_TGe136 (WT: e76<e92>e136>e176; TG: e76<e92<e136>p76). These genes’ expression is the highest at E136 in TG and at E92 in WT; Type-2: WTe136 _TGp76 (WT: e76<e92<e136>p76; TG: e76<e92<e136<p76). These genes’ expression is the highest at P76 in TG and at E136 in WT; Type-3: WTe92_TGp76 (WT: e76<e92>e136>p76; TG: e76<e92<e136<p76), these genes’ expression is the highest at P76 in TG and at E92 in WT.

***Functional enrichment analysis of the delay genes***

To test whether the identified delay genes are enriched for human specific expression genes, we assessed their enrichment scores. Human specific expression gene sets were downloaded from the previous report [20]. Enrichment was tested using the hypergeometirc probability distribution functions. The population universe was set to 19,901. *P* values were corrected by applying the Bonferroni method using the p adjust package. Enrichment was only considered significant if the Bonferroni corrected *p* value was smaller than 0.01.

***General behavioral observation***

A total of 8 monkeys including 4 wild type (WT_18–WT_21) and 4 MCPH1 transgenic monkeys (TG_05-TG_08) were included in the general behavioral observation and cognitive test. All procedures were under the guidance of IACUC of Kunming Primate Research Center, Kunming Institute of Zoology, Chinese Academy of Science (Approval No: SYDW-2010002).

Focus animal sampling was used in collecting general daily behaviors [21]. Each monkey was video-taped for two hours daily from 14:00 to 16:00 for continuous three days, and monitor was placed three days earlier before recording so that monkeys were habituated to the presence of monitor before recording. Recordings were mosaicked and named with letters were analyzed by three independent viewers. Classification and definitions of behaviors were based on previous studies [22, 23]. Briefly, self-injuring behavior including self-biting and self-hair plucking defined as severe abnormal behavior, repetitive and consistent actions were termed as stereotypical behaviors, including floating limb, self-clasping, pacing/flipping, twirling/rocking, rubbing bars, licking/biting bars and hand stereotype, and other normal daily behaviors include feeding, locomotion, self-grooming and resting.

Similar strategy was used to measure the sleep pattern of monkeys. The video system consists of two infrared video cameras facing the cages and a hard disk video recorder to collect recordings. Each monkey was taped from 18:30 – 8:30 for continuous three nights, and the recording device were placed three days before sampling. Mosaicked videos were analyzed by three independent viewers, all of them were blinded to the monkeys’ information. Sampling method was previously described in baboons’ ethology research [24]. Awake and sleep (including transitional and relaxed sleep status) were recorded in 1 minute epochs. States lasting less than 30 seconds were not considered, between 30~59s were rounded to 1min. Monkeys were considered as awake when locomotion occurred or over 3 times body movement within 1 min while in sitting or lying position, and sleep was scored as transitional when 1 or 2 times of body or limb movement occurred within 1min, relaxed sleep was defined as monkeys always kept the head below the shoulders or bent backward with no body or limb movements when sitting or lying.

***Cognitive test using delayed matching to sample task***

Cognitive tests were conducted by a non-human primate computerized touch-screen behavioral battery (Cambridge Neuropsychological Test Automated Batteries, CANTAB; Lafayette, USA) in a separated testing room (Fig. S16A). Monkeys were transfered to the testing cage (80cm high * 60cm wide * 70cm deep) in the testing room using the monkey bar, and the testing cage was modified to ensure monkeys easily touching the screen or getting reward from the CANTAB in front of it. The cognitive test procedures followed the Monkey CANTAB Manual (v1.5) and previous studies [25-27]. During the test, light was off to avoid visual noise, and an infrared camera was used to monitoring monkey status and performance during the test. Monkeys were subject to testing from 9:00 to 17:00 Monday to Friday, and a purified ingredient precision non-human primate tablet (190mg, banana taste; TestDiet, USA) were utilized in the test as food reward to minimize the need for dietary regulation and highly palatable food rewards (e.g., peanuts, raisins, or apple pieces) were given when monkey finished daily experimental session. During the whole cognitive testing period, monkeys were fed sufficient primate diet to maintain healthy. When monkeys lost > 10% weight or had diarrhea, the test would be stopped till they recovered. Water was *ad libitum,* and fresh fruits and vegetables were given after daily session.

Monkeys need to habituate to the testing environment at the beginning. Monkeys were transferred to the testing cage for 20 minutes, and apple pieces and banana taste primate tablets were given by experimenter every 5 minutes, and the light were turned off at 11^th^ min. When monkeys can get rewards naturally and anxious behaviors were rarely observed, the habituation period ends. The CANTAB was then introduced for initial shaping. At the beginning, a green square fill the touch-screen, each touch was reinforced with one banana taste primate tablet and 5-seconds blank screen inter-trial interval (ITI). Once monkeys learned the correlation, the green square became smaller after 5 effective consecutive touches, and the final square size was 120 dpi *120 dpi. Each session consists of 60 trials, which lasts about half an hour. At the Phase-II touch-training, incorrect touch or none touch within 30 seconds were regarded as incorrect or missing, and monkeys cannot get reward after an incorrect touch or missing, and an additional 5s darkness punishment (the CANTAB house light off) (Fig. S16B). During this training period, the colored square was kept as 120 dpi * 120 dpi. When monkeys reached >85% correction rate for continuous 3 sessions, they were considered passing the learning phases.

Delayed matching to sample paradigm (DMS) is analogy to Wisconsin General Test Apparatus (WGTA), a commonly used paradigm for learning test in primates. The monkeys have to touch the same stimuli at the matching phase with cue phase after delayed time. Monkeys were trained to perform the DMS task with short delayed times (0~4s) at the beginning, and when they reached the criterion (correction rate of all delays is > 75% or the session lasts for a month), they enter the next phase with increased delayed times. The terminal conditions consisted of 0s, 8s, 16s, 32s delays, each for 15 times (a total of 60 trials). The DMS stage settings are shown in Fig. S16C, and each delay was presented in a pseudorandom fashion across the total 60 trials per session in according to the CANTAB manual. The stimulus using in DMS consisted of unique patterns of four rectangular quadrants monochrome mixed shape discrimination. Data were analyzed in terms of the number of correct trials/ total trials and 5 sessions averages data were calculated for percent correct trials at each delay.

***Statistical analyses***

For MRI, the effect of age, group, feeding and age-by-group interactions of FA, MD and volumes were assessed using a linear mixed model with the following equation: dependent variable (DTI parameter or volume) = Intercept + A*age+ B*feeding+ C*age^2^+D*group+ E* age×group + F*age^2^×group. This statistical approach was chosen for its ability to handle datasets with repeated measures and varying interval between measurements. For DMS, the effect of group, session and session-by-group interactions of DMS score were assessed using a general linear model(GLM) with the following equation: dependent variable (DMS) = Intercept +group + session + group×session+ random error. The group effect p values by corrections for multiple tests were conducted using Bonferroni. R version 3.3.2 (<https://www.r-project.org/>) and R Studio (<https://www.rstudio.com/>) were used for the analysis.

***Data and software availability***

The accession number for the RNAseq data in this paper is CRA000343 (Genome Sequence Archive, GSA).

**References**

1. Fluckiger AC, Marcy G, Marchand M, et al.; Cell cycle features of primate embryonic stem cells (vol 24, pg 547, 2006). *Stem Cells* 2006;**24**(7):1832-1832. doi: DOI 10.1634/stemcells.2005-0194.

2. Niu YY, Yu Y, Bernat A, et al.; Transgenic rhesus monkeys produced by gene transfer into early-cleavage-stage embryos using a simian immunodeficiency virus-based vector. *Proceedings of the National Academy of Sciences of the United States of America* 2010;**107**(41):17663-17667. doi: DOI 10.1073/pnas.1006563107.

3. Liu Z, Li X, Zhang JT, et al.; Autism-like behaviours and germline transmission in transgenic monkeys overexpressing MeCP2. *Nature* 2016;**530**(7588):98-102. doi: 10.1038/nature16533.

4. Katz JP, Pipas JM; SummonChimera infers integrated viral genomes with nucleotide precision from NGS data. *BMC Bioinformatics* 2014;**15**:348. doi: 10.1186/s12859-014-0348-4.

5. Liu C, Tian X, Liu H, et al.; Rhesus monkey brain development during late infancy and the effect of phencyclidine: a longitudinal MRI and DTI study. *Neuroimage* 2015;**107**:65-75. doi: 10.1016/j.neuroimage.2014.11.056.

6. Zhang Y, Brady M, Smith S; Segmentation of brain MR images through a hidden Markov random field model and the expectation-maximization algorithm. *Medical imaging, IEEE transactions on* 2001;**20**(1):45-57.

7. Smith SM, Jenkinson M, Woolrich MW, et al.; Advances in functional and structural MR image analysis and implementation as FSL. *Neuroimage* 2004;**23 Suppl 1**:S208-19. doi: 10.1016/j.neuroimage.2004.07.051.

8. Das SR, Avants BB, Grossman M, et al.; Registration based cortical thickness measurement. *Neuroimage* 2009;**45**(3):867-79. doi: 10.1016/j.neuroimage.2008.12.016.

9. Avants BB, Tustison NJ, Song G, et al.; A reproducible evaluation of ANTs similarity metric performance in brain image registration. *Neuroimage* 2011;**54**(3):2033-44. doi: 10.1016/j.neuroimage.2010.09.025.

10. Jenkinson M, Beckmann CF, Behrens TE, et al.; Fsl. *Neuroimage* 2012;**62**(2):782-90. doi: 10.1016/j.neuroimage.2011.09.015.

11. Zhang H, Avants BB, Yushkevich PA, et al.; High-dimensional spatial normalization of diffusion tensor images improves the detection of white matter differences: an example study using amyotrophic lateral sclerosis. *IEEE Trans Med Imaging* 2007;**26**(11):1585-97. doi: 10.1109/TMI.2007.906784.

12. Zhang S, Peng H, Dawe RJ, et al.; Enhanced ICBM diffusion tensor template of the human brain. *Neuroimage* 2011;**54**(2):974-84. doi: 10.1016/j.neuroimage.2010.09.008.

13. Wang Y, Gupta A, Liu Z, et al.; DTI registration in atlas based fiber analysis of infantile Krabbe disease. *Neuroimage* 2011;**55**(4):1577-86. doi: 10.1016/j.neuroimage.2011.01.038.

14. Adluru N, Zhang H, Fox AS, et al.; A diffusion tensor brain template for rhesus macaques. *Neuroimage* 2012;**59**(1):306-18. doi: 10.1016/j.neuroimage.2011.07.029.

15. Zakszewski E, Adluru N, Tromp do PM, et al.; A diffusion-tensor-based white matter atlas for rhesus macaques. *PLoS One* 2014;**9**(9):e107398. doi: 10.1371/journal.pone.0107398.

16. Pertea M, Kim D, Pertea GM, et al.; Transcript-level expression analysis of RNA-seq experiments with HISAT, StringTie and Ballgown. *Nat Protoc* 2016;**11**(9):1650-67. doi: 10.1038/nprot.2016.095.

17. Trapnell C, Hendrickson DG, Sauvageau M, et al.; Differential analysis of gene regulation at transcript resolution with RNA-seq. *Nature Biotechnology* 2013;**31**(1):46-+. doi: Doi 10.1038/Nbt.2450.

18. Anders S, Huber W; Differential expression analysis for sequence count data. *Genome Biol* 2010;**11**(10):R106. doi: 10.1186/gb-2010-11-10-r106.

19. Huang da W, Sherman BT, Lempicki RA; Bioinformatics enrichment tools: paths toward the comprehensive functional analysis of large gene lists. *Nucleic Acids Res* 2009;**37**(1):1-13. doi: 10.1093/nar/gkn923.

20. Liu X, Somel M, Tang L, et al.; Extension of cortical synaptic development distinguishes humans from chimpanzees and macaques. *Genome Res* 2012;**22**(4):611-22. doi: 10.1101/gr.127324.111.

21. Altmann J; Observational study of behavior: sampling methods. *Behaviour* 1974;**49**(3):227-67.

22. Chen Y, Yu J, Niu Y, et al.; Modeling Rett Syndrome Using TALEN-Edited MECP2 Mutant Cynomolgus Monkeys. *Cell* 2017;**169**(5):945-955 e10. doi: 10.1016/j.cell.2017.04.035.

23. Walsh S, Bramblett CA, Alford PL; A Vocabulary of Abnormal Behaviors in Restrictively Reared Chimpanzees. *American Journal of Primatology* 1982;**3**(1-4):315-319. doi: DOI 10.1002/ajp.1350030131.

24. Noser R, Gygax L, Tobler I; Sleep and social status in captive gelada baboons (Theropithecus gelada). *Behav Brain Res* 2003;**147**(1-2):9-15.

25. Weed MR, Taffe MA, Polis I, et al.; Performance norms for a rhesus monkey neuropsychological testing battery: acquisition and long-term performance. *Brain Res Cogn Brain Res* 1999;**8**(3):185-201.

26. Taffe MA, Weed MR, Gutierrez T, et al.; Modeling a task that is sensitive to dementia of the Alzheimer's type: individual differences in acquisition of a visuo-spatial paired-associate learning task in rhesus monkeys. *Behavioural Brain Research* 2004;**149**(2):123-133. doi: 10.1016/S0166-4328(03)00214-6.

27. Nagahara AH, Bernot T, Tuszynski MH; Age-related cognitive deficits in rhesus monkeys mirror human deficits on an automated test battery. *Neurobiol Aging* 2010;**31**(6):1020-31. doi: 10.1016/j.neurobiolaging.2008.07.007.
